# Genomic and transcriptomic profiling of radioresistant prostate and head and neck cancers implicate a BAHD1-dependent modification of DNA damage at the heterochromatin

**DOI:** 10.1038/s41419-024-07316-y

**Published:** 2024-12-24

**Authors:** Chaw Yee Beh, Celestia Pei Xuan Yeo, Boon Hao Hong, Evelyn Mui Cheng Tan, Kah Min Tan, Dennis Jun Jie Poon, Pek Lim Chu, Dewi Susanti, Pei Ling Tai, Monica Ryu, James Proudfoot, Eugenia Li Ling Yeo, Khee Chee Soo, Melvin L. K. Chua

**Affiliations:** 1 Division of Medical Sciences, National Cancer Centre Singapore, 30 Hospital Blvd, 168583 Singapore, Singapore; 2 Veracyte, Inc, San Diego, CA USA; 3 Division of Radiation Oncology, National Cancer Centre Singapore, 30 Hospital Blvd, Singapore, 168583 Singapore; 4 Oncology Academic Programme, Duke-NUS Medical School, 8 College Road, 169857 Singapore, Singapore

**Keywords:** Translational research, Cancer models

## Abstract

Radiotherapy is an integral modality in treating human cancers, but radioresistance remains a clinical challenge due to the involvement of multiple intrinsic cellular and extrinsic tumour microenvironment factors that govern radiosensitivity. To study the intrinsic factors that are associated with cancer radioresistance, we established 4 radioresistant prostate (22Rv1 and DU145) and head and neck cancer (FaDu and HK1) models by irradiating their wild-type parentals to 90 Gy, mimicking the fractionated radiotherapy schema that is often using in the clinic, and performed whole exome and transcriptome sequencing of the radioresistant and wild-type models. Comparative genomic analyses detected the enrichment of mismatch repair mutational signatures (SBS6, 14, 15, 20) across all the cell lines and several non-synonymous single nucleotide variants involved in pro-survival pathways. Despite significant inter-cell type heterogeneity of their transcriptomic profiles, 18 common dysregulated genes (5 upregulated and 13 downregulated) were identified across the 4 models, including the overexpression of bromo-adjacent homology domain containing 1 (*BAHD1*) gene, which is involved in heterochromatin formation. Interestingly, this coincided with our observation of increased histone 3 lysine 9 trimethylation (H3K9me3) and histone 3 lysine 27 trimethylation (H3K27me3) expression post-irradiation in our radioresistant cells. The dependency between BAHD1 and heterochromatin formation was confirmed by siRNA knockdown of BAHD1, indicating preferential reduction of H3K9me3 and H3K27me3 expression in the radioresistant cells, but not the wild-type parentals, and confirmed by clonogenic assays showing reversal of radioresistance post-siBAHD1 treatment. We further showed that inhibition of the BAHD1-heterochromatin formation axis led to reduced DNA double-strand break repair. Finally, analyses of treatment outcomes in 4 prostate and head and neck cancer radiotherapy cohorts suggested an increased risk of failures in tumours of high heterochromatin activity. Taken together, our results support a new model implicating BAHD1-dependent modulation of the heterochromatin in acquired radioresistance of prostate and head and neck cancers.

## Introduction

Radiotherapy (RT) is an integral modality in the treatment of human cancers, with over 50% of cancer patients requiring RT during their treatment journey [1]. In the clinic, RT can be deployed in combination with surgery, or as a definitive treatment for organ preservation [2–4]. The conventional dogma of how RT exerts its anti-cancer effects is through either direct DNA damage from ionisation radiation or indirectly through production of reactive oxygen species [5]. However, current evidence suggests that radiosensitivity of cancers is dependent on several pathways including apoptosis, necrosis, autophagy, senescence, and more recently, immunogenic cell death mechanisms like necroptosis and ferroptosis [6–9].

Factors like the oxygenation status of the tumour microenvironment (TME) also influence the efficacy of RT on the cancer cell [10]. Separately, the cancer cell can manifest intrinsic radioresistance during the course of RT, through accommodation of stress signals and upregulation of DNA repair pathways [11]. The latter model would represent the concept of acquired radioresistance, which is often observed in the clinic. For example, patients with advanced head and neck cancer often develop locoregional recurrences in the same region after a long course of fractionated high dose RT [12].

**Fig. 1:**
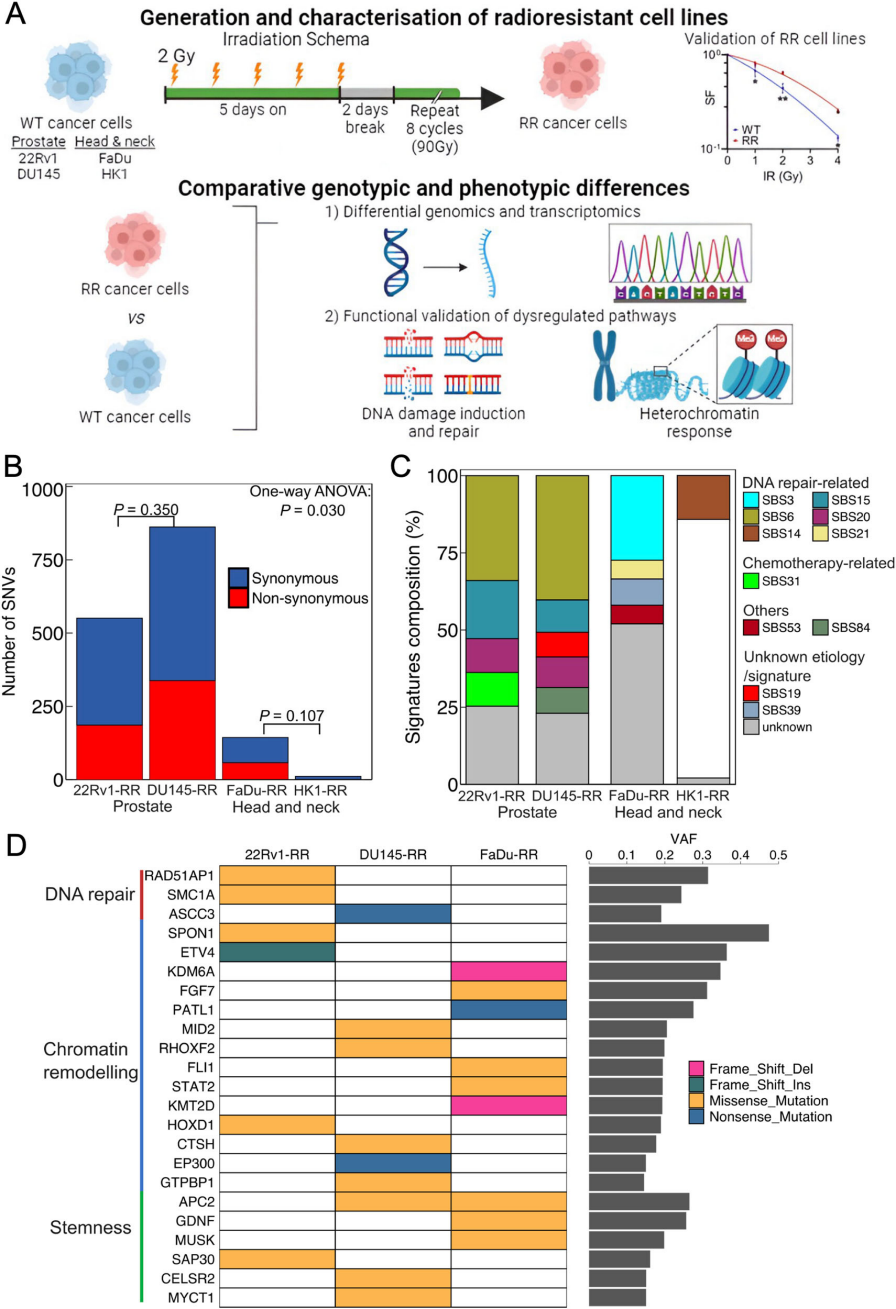
Genomic profiling of RR prostate and head and neck cancer cells. **A** Schema of generation and characterisation of radioresistant cell lines with downstream experiment plan (created with Biorender.com). **B** Total counts of SNV for four RR cell lines included synonymous and non-synonymous SNV. **C** Distribution of COSMIC mutational signatures identified by all SNVs in each cell line. Only SBS14 is shown for HK1, as other mutational signatures were removed due to overfitting caused by the limited number of SNVs in HK1. **D** The annotated non-synonymous SNV grouped by the related function in DNA repair, chromatin remodelling, and stemness pathways curated from Gene Ontology libraries. RR radioresistant, SNV single nucleotide variant.

To study the cellular intrinsic factors underpinning radioresistance of human cancers, we had established a panel of radioresistant (RR) prostate and head and neck cancer models by high-dose X-irradiation (90 Gy) of the parental wild-type (WT) cell lines. We simulated the treatment schema to mimic the delivery of conventional fractionated RT (1.8–2.0 Gy per daily-fraction) that is classically used in the clinic, albeit we allowed for longer treatment breaks between weekly courses of irradiation (IR) (Fig. 1A). We first performed whole exome and transcriptome sequencing to characterise the differential genomic and transcriptomic profiles between the RR and WT cancer models. These results revealed the heterochromatin response as a common mechanism of radioresistance in our prostate and head and neck cancer models, and we further delineated BAHD1 as a potential target that is implicated in driving the enhanced heterochromatin response. Finally, we posit that the BAHD1-dependent pathway influences the radiosensitivity of our cancer models by modulation of DNA repair, thereby providing a new model that is implicated in cancer radioresistance.

## Results

### Genomic alterations of RR prostate and head and neck cancer cells

We investigated the genomic alterations that were enriched in our RR cancer models by comparing the genomic profiles of the RR cells against their WT cells. To this end, mutations that occurred in 2 of the triplicates were counted. Overall, we observed a higher single nucleotide variant (SNV) count in our RR prostate cancer cells compared with RR head and neck cancer cells (Fig. 1B). Among the 4 cancer models, we observed that DU145-RR cells possessed the highest number of acquired SNVs, whereas HK1-RR cells possessed the lowest number of acquired SNVs (SNV counts: 862 [DU145-RR] vs 551 [22Rv1-RR] vs 144 [FaDu-RR] vs 11 [HK1-RR], *P* = 0.030, one-way ANOVA). Additionally, most of the observed SNVs were synonymous mutations, indicating that these were likely random passenger mutations.

For COSMIC mutational signatures, we observed homogeneity in the distribution of mutational signatures between the RR prostate cancer models, whereas mutational signatures varied between prostate and head and neck models, and further differed between FaDu-RR and HK1-RR cells (Fig. 1C). Specifically, the RR prostate cancer models were dominated by DNA repair-specific signatures (SBS6, SBS15, and SBS20), contrasting with the RR head and neck cancer models that manifested mostly non-DNA repair mutational signatures (Supplementary Table 1). Nonetheless, we identified the presence of DNA mismatch repair (MMR)-related mutational signatures (SBS3, SBS14, and SBS21) across the 4 RR cancer cell lines.

To investigate the role of the non-synonymous SNVs in the RR cancer cells, we proceeded to annotate the functions of the top 30 frequently mutated genes using the Gene Ontology (GO) library (Fig. 1D). Of note, we observed only 1/11 non-synonymous SNVs (*KLHL38*) related to protein binding in HK1-RR cells, which is consistent with the low number of acquired SNVs. Next, SNVs in DNA repair genes (RAD51-associated protein 1 [*RAD51* *AP1*], structural maintenance of chromosomes 1A [*SMC1A*], and activating signal cointegrator 1 complex subunit 3 [*ASCC3*]) were observed only in the RR prostate cancer cells. Interestingly, we found several SNVs related to chromatin remodelling and cancer cell stemness in 22Rv1-RR, DU145-RR, and FaDu-RR cells. Of note, adenomatous polyposis coli protein 2 (*APC2*), which plays a role in the WNT signalling pathway, was mutated in both DU145-RR and FaDu-RR cells. Taken together, these findings reveal the presence of several pro-survival genotypic features in our RR cancer models following a course of 90 Gy fractionated IR.

### Transcriptomic profiling of RR prostate and head and neck cancer cells

**Fig. 2:**
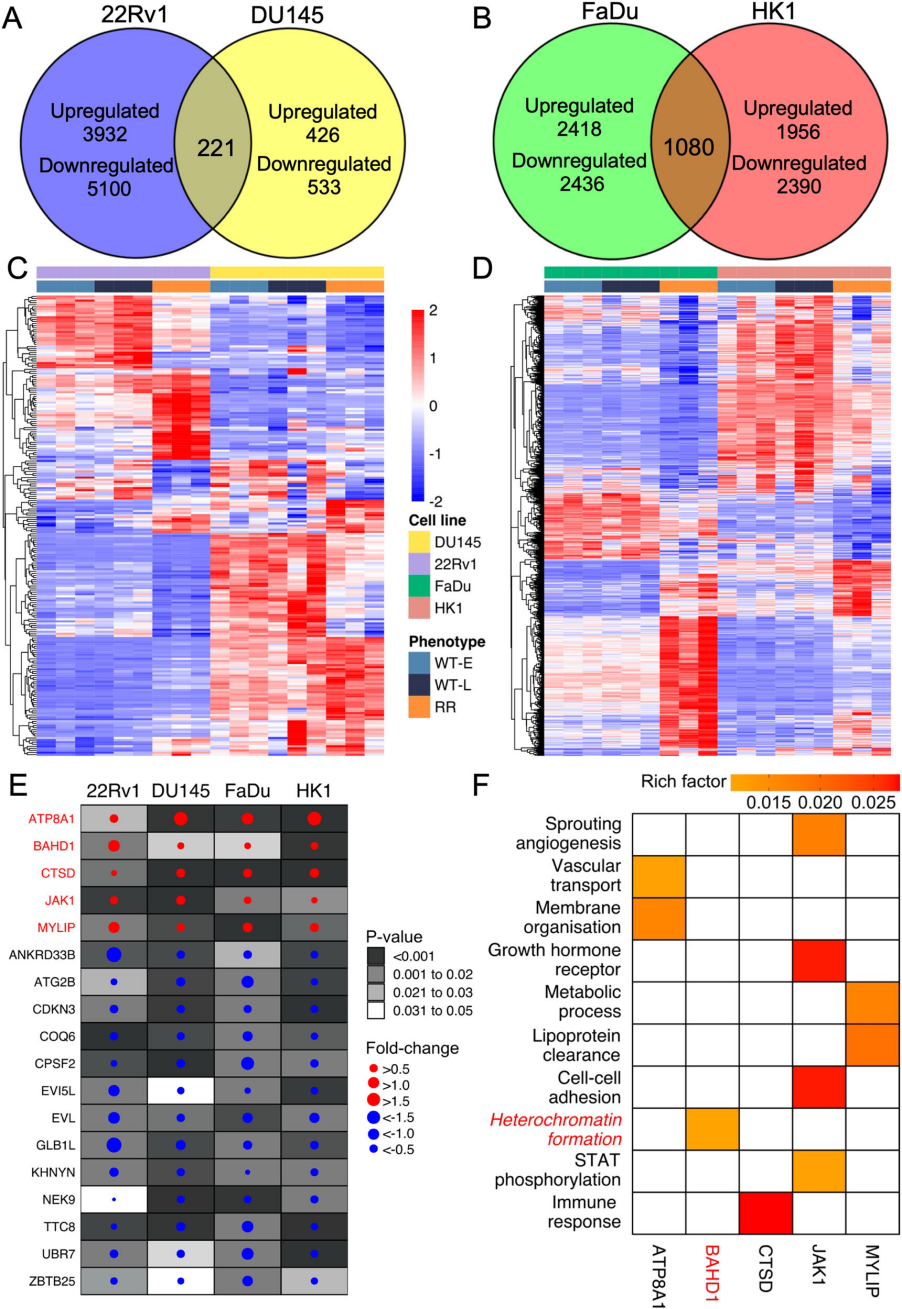
Transcriptome profiling of RR prostate and head and neck cancer cells. **A** Differentially expressed genes (DEGs) in 22Rv1 and DU145, and (**B**) in FaDu and DU145. The DEGs were defined if the adjusted *P* < 0.05. **C** Expression profile of 221 common DEGs with consistent dysregulation direction in both 22Rv1 and DU145. **D** Expression profile of 1420 common DEGs with consistent dysregulation direction in both FaDu and HK1. Samples were ordered by cell lines and phenotype in both figures. **E** 18 DEGs with consistent dysregulation direction across four cell lines. The colour of each dot indicates the dysregulation direction, with red indicating upregulation and blue indicating downregulation. The size of each dot varies based on its fold change. The intensity of colour within each box reflects the range of *P*-values. **F** Significant pathways involved by the 5 upregulated DEGs across the 4 cell lines, curated from over-representation analysis (false discovery rate <0.1). The colour intensity within each colour filled box represents the rich factor of the gene in the pathway.

Comparative transcriptomic analyses revealed several dysregulated genes between the RR and WT cells (median of 4597, interquartile range [IQR]: 3499–5644 across the 4 cell lines; 2187 were upregulated and 2410 were downregulated, see Supplementary Fig. S1 for analyses of each cell line). Dysregulated genes that intersected between the pairs of prostate (22Rv1 and DU145) and head and neck cancer cells (FaDu and HK1) were considered for subsequent analyses; this led to the selection of 221 (117 up and 104 down) and 1,080 (458 up and 622 down) affected genes in the RR prostate and head and neck cancer models, respectively (Fig. 2A, B).

Next, unsupervised clustering and principal component analyses of the mRNA abundance of these genes indicated substantial inter-cell type heterogeneity between 22Rv1 and DU145, as well as between FaDu and HK1, independent of their passage status (Fig. 2C, D, Supplementary Fig. S2). Magnitudes of fold-change also differed for the affected genes, with 20/221 (9.1%) and 62/1080 (5.7%) of the common genes manifesting >1 fold-change for the prostate and head and neck cancer models, respectively. Based on the acquired mutational signatures observed in the RR models (Fig. 1C), we further investigated for differential gene expression of 548 genes related to DNA repair between the RR and WT cells (GO:0006281, Supplementary Fig. S3). Of note, we did not observe any significant difference in mRNA abundance of DNA repair genes between the RR and WT cells.

Regardless of heterogeneity in gene expression, we were able to identify 18 genes that were uniformly affected (5 upregulated and 13 downregulated) across the 4 RR cancer cell lines (Fig. 2E). We then hypothesised that the 5 upregulated genes (namely, ATPase phospholipid transporting 8A1 [*ATP8A1*], *BAHD1*, cathepsin [*CTSD*], janus kinase 1 [*JAK1*], and myosin regulatory light chain interacting protein [*MYLIP*]) play a role in radioresistance of prostate and head and neck cancers.

To further delineate their roles in conferring an RR phenotype, we performed an over-representation analysis of the 5 upregulated genes [13], whereby we tested the weightage of these genes in their respective GO pathways (Fig. 2F, Supplementary Table 2). We identified 10 significant GO pathways that spanned from metabolic processes, immune response, cell-cell adhesion, to heterochromatin formation.

Of note, heterochromatin formation (GO:0031507) is linked to *BAHD1* [14], which is a heterochromatin-associated protein that recruits co-repressors and binds to H3K27me3, thereby suppressing histone acetylation at its Polycomb gene targets. Given the published literature linking the heterochromatin response to radiation effects in cancer cells [15], we chose to focus on elucidating the role of BAHD1 in modulating H3K27me3 and H3K9me3 expression following IR of our 22Rv1- and FaDu-RR and -WT cells.

### DNA damage and heterochromatin responses of 22Rv1-RR and FaDu-RR cancer cells

**Fig. 3:**
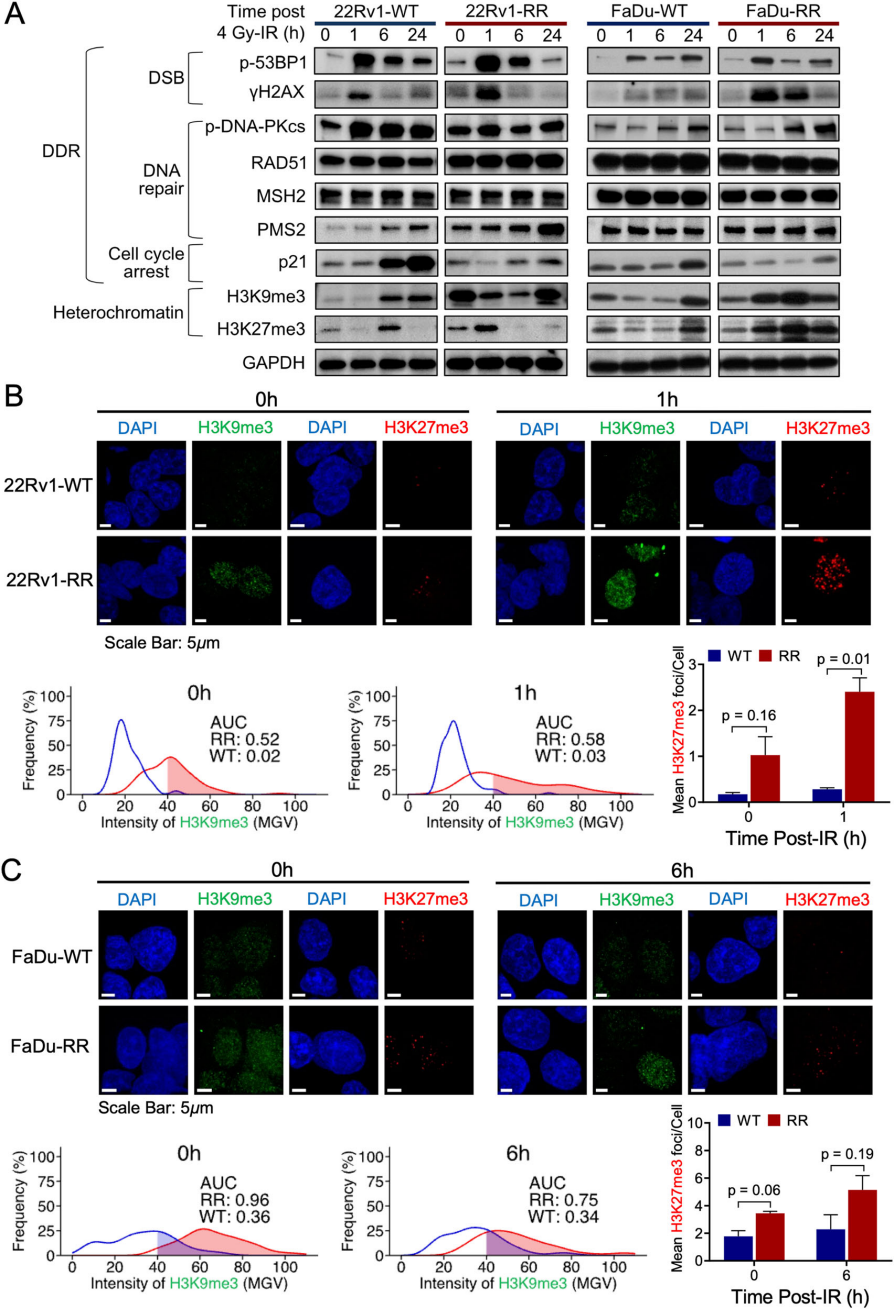
Characterisation of the DNA damage responses and heterochromatin status of 22Rv1- and FaDu-RR cells relative to the parental WT cells. **A** The representative western blot showed the changes in the expression of DSB, DNA repair, cell cycle arrest and heterochromatin markers under different time points post-4 Gy IR (normalised against control), GAPDH was used as a loading control. **B and C-top panel** Representative images of H3K9me3 (green) and H3K27me3 (red) foci in 22Rv1 cells at 0 and 1 h treatment time points and FaDu cells at 0 and 6 h treatment time points. Scale bar: 5 μm. **B and C-bottom panel** H3K9me3 intensity was quantified in AUCs and represented as percentage frequencies, compared between RR and WT; H3K27me3 foci were quantified as mean foci per cell, bars represent mean±SD, *n* = 3 per group. DSB DNA double-strand break, AUC area under the curve, WT wild-type, SD standard deviation.

Given the genomic findings indicating the presence of DNA repair mutational signatures and non-synonymous SNVs affecting DNA repair genes in our RR cancer models, we characterised the DNA double-strand break (DSB) repair efficiency of 22Rv1-RR and FaDu-RR cells compared with the WT parentals following 4 Gy IR. Semi-qualitative and quantitative assessment of γH2AX and p-53BP1 expression by western blot (WB) and foci immunofluorescence (IF) assays, respectively, did not reveal consistent observations in DSB induction and repair kinetics between 22Rv1 and FaDu cells. For 22Rv1, while DSB induction was comparable between the RR and WT cells, we observed an enhanced repair efficiency at 6 and 24 h in the former (Fig. 3A, Supplementary Fig. S4A), whereas we did not observe differences in both DSB induction and repair kinetics for FaDu cells (Supplementary Fig. S4B). This corresponded to an increased expression of RAD51 recombinase (RAD51) at 6 and 24 h post-IR for 22Rv1-RR cells, although phosphorylated DNA protein kinase catalytic subunit (p-DNA-PKcs) expression did not differ between the RR and WT cells across the time points. We performed a detailed analyses of the different proteins involved in MMR pathway since we observed enrichment of MMR mutational signatures in our RR cancer models. Interestingly, we observed higher expression of MSH6 and PMS2 in 22Rv1-RR than -WT cells pre- and post-IR, but not for FaDu-RR and -WT cells, thereby providing a mechanistic linkage between our mutational signature results and downstream protein expression at least for 22Rv1 (Fig. 3A, Supplementary Fig. S5). Simultaneously, p21 expression was reduced for 22Rv1-RR and FaDu-RR compared with the WT cells. Taken together, apart from enhanced DSB repair kinetics that may be attributed to homologous recombination, along with increased MMR activity in 22Rv1-RR cells, and possibly compromised cell cycle checkpoint responses in both 22Rv1-RR and FaDu-RR cells, we did not observe substantial differences in the hallmarks of DNA damage response (DDR) between our RR and WT models.

To understand the link between BAHD1 gene overexpression and heterochromatin response to IR in our RR models, we first assessed the expression of two heterochromatin markers (H3K9me3 and H3K27me3) by WB and IF assays in our RR and WT cells post-4 Gy IR. We observed higher expression of both heterochromatin markers on WB for 22Rv1-RR and FaDu-RR than -WT cells post-IR, although both markers were also overexpressed for 22Rv1-RR cells pre-IR (Fig. 3A). This corresponded to our IF analyses showing an increased H3K27me3 foci counts pre-IR and at 1 h post-IR in 22Rv1-RR cells (mean count/cell of 1.02 vs 0.17, Student’s T *P* = 0.16 [0 h]; 2.40 vs 0.28, *P* = 0.01 [1 h]), but not at 6 h and 24 h (Fig. 3B, Supplementary Fig. S6A). To quantify the difference in H3K9me3 intensity on IF, we derived area under the curve (AUC) values for the probability densities of RR and WT cells that displayed >40 mean gray value (MGV) (Fig. 3B, Supplementary Fig. S7); AUC values of cells with >40 MGV were higher in the 22Rv1-RR cells than -WT cells pre-IR (0.52 vs 0.02) and at 1 h post-IR (0.58 vs 0.03).

Similarly, in our FaDu cells, we observed an increased H3K9me3 intensity and H3K27me3 foci counts pre-IR and at 6 h post-IR for the RR cells, but not at 1 h and 24 h; AUC values of H3K9me3 intensity were 0.96 versus 0.36 pre-IR and 0.75 versus 0.34 at 6 h post-IR, and corresponding mean H3K27me3 foci counts were 3.45 versus 1.77 (*P* = 0.06) and 5.14 versus 2.28 (*P* = 0.19) (Fig. 3C, Supplementary Fig. S6B). Collectively, our data suggests distinct differential heterochromatin responses post-IR between our RR and WT cancer cells, thereby supporting the results of our transcriptomic analyses.

### Knockdown of BAHD1 reversed the enhanced heterochromatin response and radioresistance in 22Rv1 and FaDu RR cells

**Fig. 4:**
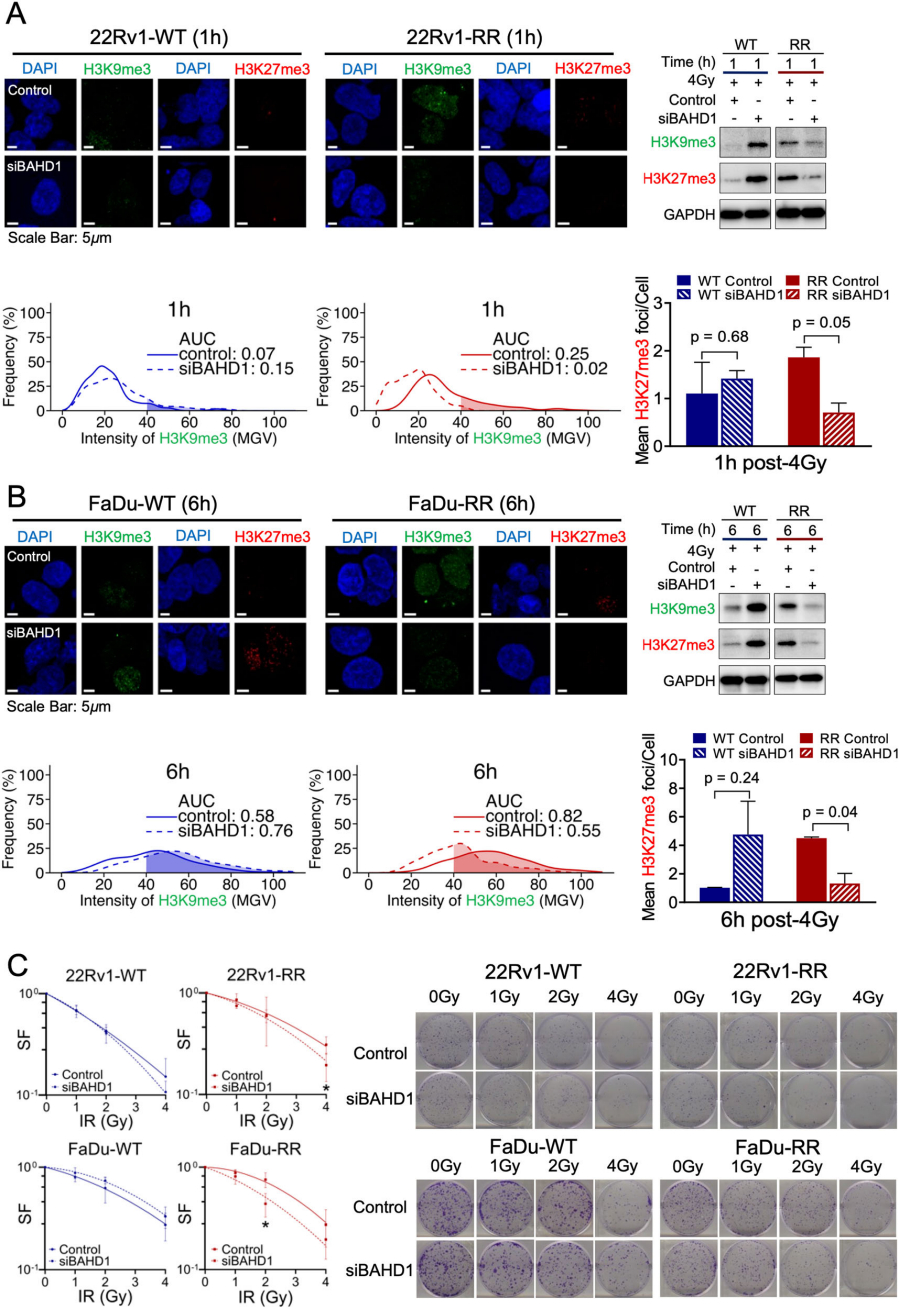
The heterochromatin status with and without siBAHD1 treated-22Rv1-RR and FaDu-RR cells were relative to the parental WT cells, and their clonogenic survivability. **A and B-top left** Representative images of H3K9me3 (green) and H3K27me3 (red) foci in 22Rv1 cells at 1 h and FaDu cells at 6 h post-siBAHD1 treatment. Scale bar: 5 μm; (top right) Representative western blot showed the changes in H3K9me3 and H3K27me3 protein levels in 22Rv1 cells at 1 h and FaDu cells at 6 h post-siBAHD1 treatment; (bottom panel) H3K9me3 intensity was quantified in AUCs and represented as percentage frequencies, compared between RR and WT; H3K27me3 foci were quantified as mean foci per cell, bars represent mean±SD, *n* = 3 per group. **C** Colony forming assay of 22Rv1 and FaDu cells under different IR dosages, with and without siBAHD1 treatment, *n* = 3 per group. Asterisk indicates significance between siBAHD1-treated and control groups. Asterisk indicates *P* < 0.05.

To confirm the involvement of BAHD1 in modulating the expression of H3K9me3 and H3K27me3, we next performed functional knockdown experiments of BAHD1 using the siRNA approach in both cancer models (Supplementary Fig. S8). First, we assessed the relative expression of both heterochromatin markers in our siBAHD1-treated 22Rv1 and FaDu RR and WT cells after 4 Gy IR. WB and IF analyses showed reduced H3K9me3 intensity and H3K27me3 foci counts post-siBAHD1 treatment in our 22Rv1-RR cells at 1 h (AUC: 0.02 [siBAHD1] vs 0.25; mean count/cell: 0.70 [siBAHD1] vs 1.86, *P* = 0.05, Fig. 4A), and FaDu-RR cells at 6 h (AUC: 0.55 [siBAHD1] vs 0.82; mean count/cell: 1.32 [siBAHD1] vs 4.49, *P* = 0.04, Fig. 4B) post-IR. This phenomenon was however not observed in the WT cells with siBAHD1 treatment (Supplementary Figs. S9, S10). Our data thus suggest the mechanistic linkage between BAHD1 and the enhanced heterochromatin response in our RR cancer models.

Next, we repeated the clonogenic forming assays in 22Rv1-RR and FaDu-RR cells with and without siBAHD1 treatment. While treatment with siBAHD1 did not influence the cellular radiosensitivity of WT cells (SF_2Gy_ siBAHD1:control=0.87 [FaDu-WT]; SF_4Gy_ siBAHD1:control=0.88 [22Rv1-WT]), both 22Rv1-RR and FaDu-RR cells manifested an increased radiosensitivity post-siBAHD1 treatment, (SF_2Gy_ siBAHD1:control=1.72 [FaDu-RR], SF_4Gy_ siBAHD1:control=1.63 [22Rv1-RR], Fig. 4C). Taken together, our findings support the notion that BAHD1 overexpression is implicated in the radioresistance of our prostate and head and neck cancer models through modulation of the heterochromatin response to IR.

### Inhibition of BAHD1-dependent heterochromatin response increases DSB induction and impairs repair in 22Rv1- and FaDu-RR cells

**Fig. 5:**
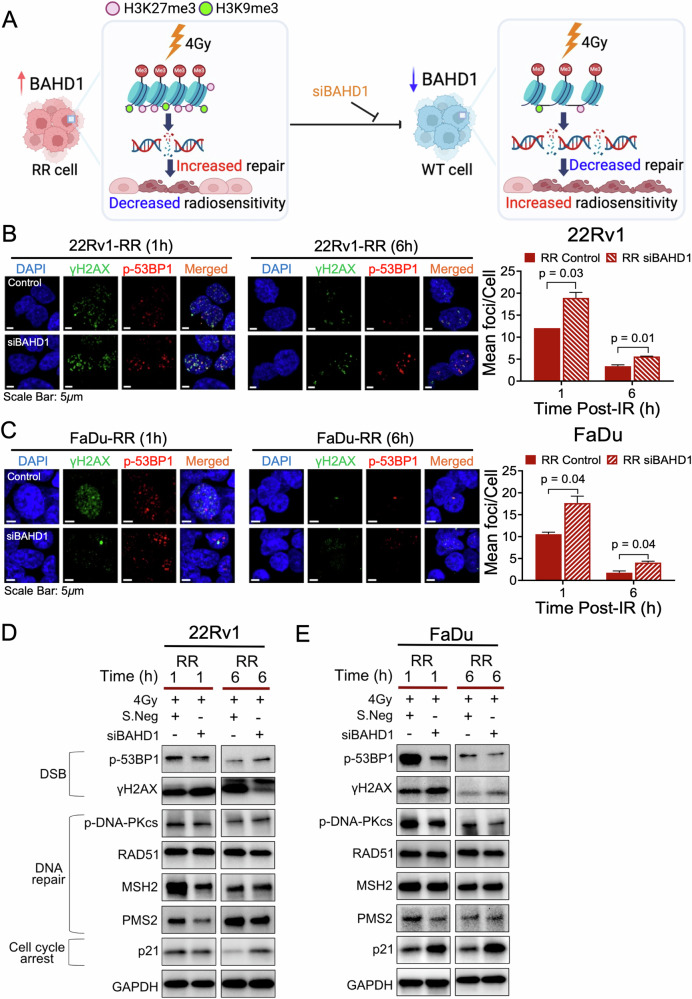
Reduced BAHD1 expression improved the radiosensitivity of 22Rv1-RR and FaDu-RR cells relative to the parental WT cells. **A** A hypothesised model showing BAHD1 contributes to the enhanced heterochromatin response in RR cancer cells, whereby increased repair efficiency following irradiation (IR)-induced damage leads to decreased radiosensitivity. The inhibition of BAHD1 leads to chromatin unpacking, decreased repair efficiency and improved radiosensitivity (created with Biorender.com). **B and C, left** Representative images of co-localised γH2AX (green) and p-53BP1 (red) foci in 22Rv1-RR and FaDu-RR cells at 1 and 6 h post-IR, with and without siBAHD1 treatment. Scale bar: 5 μm; (**right**) Quantification of co-localised γH2AX and p-53BP1 mean foci per cell, bars represent mean±SD, *n* = 3 per group. **D, E** Representative western blot showed the changes in the expression of DSB, DNA repair, cell cycle arrest and heterochromatin markers of 22Rv1-RR cells and FaDu-RR cells at 1 and 6 h post-IR, with and without siBAHD1 treatment (normalised against control).

To further investigate the mechanisms underpinning a BAHD1-dependent enhanced heterochromatin response and radioresistance, we queried if increased expression of heterochromatin markers alters chromatin packaging, leading to enhanced DSB repair capacity. Here, we posit a model that fractionated IR delivered to 90 Gy induced a constellation of alterations at the genome and transcriptome, including BAHD1 overexpression that led to enhancement of the heterochromatin response to IR, thereby increasing the DSB repair capacity and pro-survival ability of our RR cancer cells (Fig. 5A).

To this end, we tested the DSB induction and repair capacity at 1 h and 6 h post-IR following siBAHD1 treatment in our RR models. For both 22Rv1-RR and FaDu-RR cells, we observed increased DSB induction and reduced repair post-treatment, (Fig. 5B, C, Supplementary Fig. S11). This corresponded to a lower expression of p-DNA-PKcs at 1 h and 6 h post-IR in the siBAHD1-treated FaDu-RR cells, but not for the 22Rv1-RR cells (Fig. 5D, E, Supplementary Fig. S11). Conversely, expression of MSH2 and PMS2 was decreased in 22Rv1-RR cells, but not FaDu-RR cells, at 1 h post-IR following siBAHD1 treatment. Expression of RAD51 was however not influenced by siBAHD1 treatment in both cell lines. Additionally, we observed higher p21 expression in both siBAHD1-treated 22Rv1-RR and FaDu-RR cells post-IR, which is indicative of an increased cell cycle checkpoint response. While we acknowledged that our earlier results (as shown in Fig. 3A) suggest that DSB repair capacity only differed for 22Rv1, nonetheless, we uncovered an interplay between manipulation of BAHD1 expression and DSB induction and repair in both our RR prostate and head and neck cancer cells.

### Heterochromatin overexpression is prognostic in patients with prostate cancer and head and neck cancer treated by RT

**Fig. 6:**
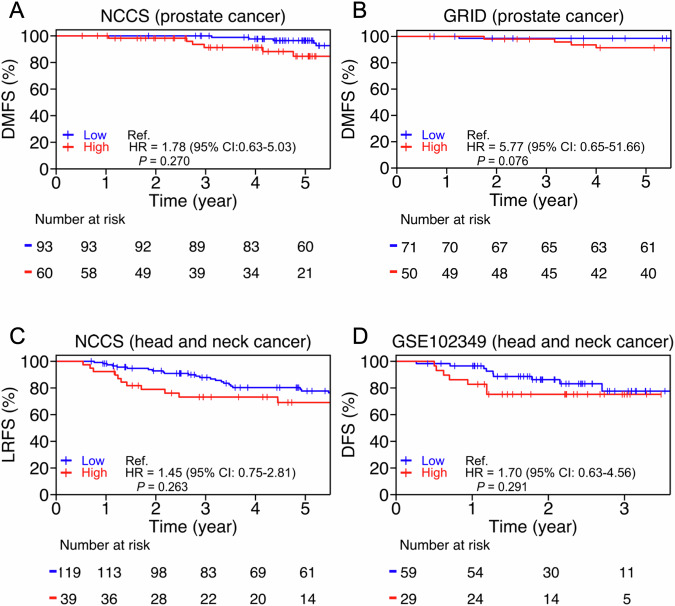
Increased heterochromatin formation associated with risk of relapse in prostate cancer and head and neck cancer patients after radiotherapy. Kaplan-Meier curves of the patients’ stratified heterochromatin activity in (**A**) NCCS prostate cancer cohort (*n* = 151); (**B**) Berlin et al. prostate cancer cohort from GRID (*n* = 121); (**C**) NCCS head and neck cancer cohort (*n* = 158); (**D**) Zhang et al. head and neck cancer (GSE102349) cohort (*n* = 88). The hazard ratios and 95% confidence intervals were computed by the COX proportional hazard model, and survival curves were compared by the log-rank test.

Finally, to support our model that an enhanced heterochromatin response is linked to cancer radioresistance, we explored the prognostic associations of heterochromatin pathway activity and outcomes in patients with prostate and head and neck cancer treated with RT. Here, we utilised 4 patient cohorts for which both clinical and transcriptomic data were available; 2 cohorts consisted of patients treated at our institution [16, 17], 1 external prostate cancer cohort published by Berlin et al. from an ongoing collaboration [18], and 1 public head and neck cancer dataset (GSE102349) [19]. We examined if high heterochromatin pathway enrichment scores were positively associated with risk of relapse post-RT. Our analyses consistently indicated that high enrichment scores of heterochromatin formation exhibited a trend for increased risk of relapse in all cohorts, thereby supporting our proposed concept of heterochromatin modification as a potential pathway of radioresistance in these cancers (Fig. 6).

## Discussion

In the clinic, it is observed that therapeutic responses to RT vary between cancer types, and between patients harbouring the same cancers despite comparable treatment regimens, thereby suggesting that clinical radiosensitivity of cancers is heterogeneous [20, 21]. To interrogate the intrinsic tumour factors underpinning radioresistance, we set up a panel of RR cancer models, focusing on prostate and head and neck cancers, that were derived following treatment with an IR schema that mimics the RT regimen often prescribed in the clinic. These models enabled us to robustly study the intrinsic cellular changes that were associated with clonal selection and adaptation leading to acquired radioresistance [22, 23]. From comparative analyses of our RR and WT cancer cells, we made several observations that were enriched in the former: (1) first, there were acquisition of several mostly synonymous SNVs that were involved in various pro-survival pathways e.g., cancer cell stemness, chromatin remodelling, etc. along with enrichment of mismatch repair mutational signatures; (2) second, we observed significant inter-cell type heterogeneity in transcriptional dysregulation between the RR and WT prostate and head and neck cancer cells, where DU145 had the lowest number of DEGs, but nevertheless, were able to identify 18 common genes that included overexpression of BAHD1; (3) coincidentally, we tied this observation to an ubiquitously enhanced heterochromatin response following IR in our 22Rv1-RR and FaDu-RR cells, and showed the dependency of this response to BAHD1 expression; (4) we then attempted to elucidate the linkage between BAHD1-dependent heterochromatin response and DDR, and showed the influence of BAHD1 on DSB induction and DNA-PKcs-dependent end-joining and MMR; (5) finally, we supported our experimental findings by showing that higher heterochromatin activity was associated with increased risk of treatment failures in patients with prostate and head and neck cancers who were treated with RT, although these analyses did not achieve statistical significance. Herein, we have demonstrated the utility of our platform that led to the discovery of a new pathway for radioresistance involving BAHD1-dependent modification of the heterochromatin that was a dominant molecular phenotype in our RR prostate and head and neck cancer models.

Our observation of an enhanced heterochromatin response in RR cancers is corroborated by a recent study in RR nasopharyngeal carcinoma, which reported the same observation of H3K27me3 overexpression [24]. Interestingly, there have been reports linking the heterochromatin and DNA repair, either by directly influencing non-homologous end-joining or indirectly via the H3K9me3-Tip60 (histone acetyltransferase KAT5)-ATM (ataxia telangiectasia mutated) signalling axis [25, 26]. For the former, Zhang et al. showed that higher expression of heterochromatin led to increased end-joining, while Sun et al. showed that depletion of H3K9me3 impaired Tip60 recognition of ATM, and consequently, downstream DDR signalling. Here, we reported an upstream mechanism whereby heterochromatin formation is increased by BAHD1 overexpression in our RR prostate and head and neck cancer models. Our results also hinted at a downstream effect of this pathway on DSB repair (knockdown of BAHD1 resulted in increased DSB in both RR models, corresponding to reduced expression of DNA-PKcs in FaDu-RR cells, and MSH2 and PMS2 in 22Rv1-RR cells). Collectively, these findings add to the existing evidence supporting the role of heterochromatin in mitigating radiosensitivity. Going forward, more work is required to elucidate the mechanisms underpinning BAHD1-dependent heterochromatin overexpression and its downstream consequences.

Some limitations of our study deserve mention. First, we acknowledged that our RR cancer models were by no means comprehensive given that they do not model the effects of the TME following a long course of IR. Nevertheless, we focused on studying the cellular changes post-IR in our panel of prostate and head and neck cancer cell lines, as previous studies had only superficially investigated the cellular effects of fractionated IR in single cancer models [27–29]. Second, we focused solely on the heterochromatin formation pathway in this study, while other genes and pathways identified from our over-representation analysis (Fig. 2F) may also contribute to radioresistance. To encourage future research, we have deposited the raw sequencing data of our WT and RR cancer cells (that were established in-house) in the public domain. Third, admittedly, downstream molecular analyses were only performed in 2 of the 4 cancer models, but this was partly due to the fewer DEGs identified in DU145 cells, and a low number of acquired mutations in HK1-RR cells. Fourth, our results do not yet provide clarity on the interplay between the heterochromatin response and DSB repair. While BAHD1 knockdown seemingly reduced the heterochromatin response and led to increased DSB induction and reduced repair in both 22Rv1-RR and FaDu-RR cells, the differences in DDR were not obvious in the baseline comparisons between the RR and WT cancer cells. Elucidation of the mechanisms underpinning BAHD1-dependent heterochromatin formation would require in-depth epigenetic analyses of the chromatin remodelling processes. Lastly, we acknowledge that the prognostic associations from the clinical analyses are suggestive at best. However, this limitation stems from the availability of public datasets for RT-treated patients with paired molecular data of their tumours. Additionally, the low recurrent event rates for the respective cohorts could have contributed to the inability to achieve statistical significance.

Herein, we undertook comparative genomic and transcriptomic analyses of a panel of RR prostate and head and neck cancer models against their parental WTs, and provided a thorough overview of the mutational features that were associated with acquired radioresistance. From these analyses, we uncovered a new heterochromatin modification pathway via BAHD1 overexpression that was a molecular driver of radioresistance in these cancers. Further understanding of BAHD1 modification of the chromatin structure to influence radiosensitivity could help to discover therapeutic targets against this molecular vulnerability of RR cancers.

## Materials and methods

### Cell lines and culturing conditions

Prostate and head and neck cancer cell lines 22Rv1, DU145, and FaDu were purchased from the American Type Culture Collection (ATCC), routinely tested for mycoplasma contamination with EZ-PCT^TM^ Mycoplasma Detection Kit (Biological Industries, USA) and authenticated using short tandem repeat analysis by ATCC (ATCC, USA). The Epstein-Barr virus (EBV)-negative nasopharyngeal carcinoma, HK1, was derived from a 41-year-old Chinese male patient with nasopharyngeal carcinoma [30] and donated by Professor George Tsao from the University of Hong Kong. Cells were cultured in either Roswell Park Memorial Institute (Gibco, New York) or Dulbecco’s Modified Eagle Medium (Gibco, New York), supplemented with 10% v/v fetal bovine serum (HyClone™, UT), 1% non-essential amino acid (Gibco, New York), 1% L-Glutamine (Gibco, New York), 1% sodium Pyruvate (Gibco, New York), and 1% penicillin-streptomycin (Gibco, New York). All cells were maintained in a humidified incubator at 37 °C with 5% CO_2_ and passaged upon reaching 80% confluency. Early passages (10 or below) were utilised for all experiments.

### Generation of RR cancer cell lines

The RR cell lines (22Rv1-RR, DU145-RR, FaDu-RR, and HK1-RR) were generated by exposing their respective parental wild-type (WT) cells to a course of fractionated 2 Gy IR over 45 daily treatments, excluding weekends, to a total dose of 90 Gy. Cells were seeded in a 175 cm^2^ flask and irradiated using a Gamma Cell® GC40 exactor source ^137^Cs-137 (Nordion, Canada) at a 0.9 Gy/min dose rate. The media of the irradiated cells were routinely changed, and cells were passaged once they reached 80% confluency.

### Colony formation assays

Clonogenic assays were performed to determine the radiosensitivity of the irradiated cells relative to their WT counterparts after a total of 90 Gy fractionated IR (Fig. 1A, Supplementary Fig. S12). Briefly, cells were cultured in a 60 mm dish and irradiated with doses of 1, 2, and 4 Gy using a gamma irradiator. The irradiated cells were then trypsinised and re-plated in 6-well plates. After 10–14 days, the cells were stained with 0.05% crystal violet for 1 h. Colonies with more than 50 cells were counted with a light microscope, *n* = 3 per group. Plating efficiency (PE) was calculated by the ratio of the number of colonies counted to the total number of plated cells. The surviving fraction (SF) was calculated by the ratio of the PE of the irradiated cells to the PE of the non-irradiated cells. Survival curves were generated using GraphPad Prism8 software (version 8.0.2 [263]) and the non-linear regression LQ model (Y = EXP[-(B1*X + B2*X^2)]).

### Whole exome sequencing

Total DNA was extracted from the cells using the QIAamp DNA Blood Mini Kit (QIAGEN, Maryland) according to the manufacturer’s instructions. WES libraries were prepared using the Agilent SureSelect Human All ExonV6 Kit (Agilent Technologies, CA), and 150 bp paired-end sequencing was performed using the Novaseq 6000 (Illumina, CA) to depths of 100X per sample (at least 10 GB per sample). The DNA reads were subsequently aligned to the hg38 reference genome. The resulting SAM files were then converted to BAM format with SAMtools. Following this, the BAM files were merged using SAMtools, and any duplicate reads were flagged and removed using MarkDuplicatesSpark, resulting in coordinate-sorted and indexed BAM files. Base quality score recalibration (BQSR) was performed using the Genomic Analysis Toolkit (GATK).

### RNA sequencing

Total RNA was extracted from RR cells using the RNeasy Mini Kit (Qiagen, Maryland) according to the manufacturer’s instructions. Stranded RNAseq libraries were prepared by poly(A) mRNA isolation and NEBNext Ultra II Directional RNA Library Prep Kit (New England BioLabs, MA). Next, 150 bp paired-end sequencing was performed using the Novaseq 6000 (Illumina, CA) with at least 50 million reads per sample. Adapter sequences and low-quality base calls were removed from the raw sequence reads using Trim Galore (v0.6.4) and Cutadapt (v2.10). Trimmed reads were then mapped to the hg38 reference genome using Spliced Transcripts Alignment to a Reference (STARv2.6.1 d) with standard settings. Gene quantification was performed using the “--quantMode GeneCounts” option in STAR.

### Variant mutation calling

Each RR cell line was matched with its respective parental WT cell lines to identify genomic mutations. Mutations were independently called using three different tools: Mutect2 (GATK v4.1.8.0), Manta (v1.6.0) with Strelka2 (v2.9.10), and Lancet (v1.1.0). The output variant call format (VCF) files from these tools were transformed into mutation annotation format (MAF) using the vcf2maf tools [31]. Sites detected by at least two of these tools and with consensus in at least duplicates were counted as variants. DeconstructSigs [32] was used to determine the COSMIC mutational signatures using the identified SNVs in each cell line. The function of non-synonymous SNVs in each cell line was annotated using GO libraries.

### Transcriptomic analyses of RR versus parental WT cells and pathway curation for gene of interest

Comparative differential gene expression analysis of RR versus parental WT cells (regardless of cell passage status) for each cell line was conducted independently using DESeq2 (v1.38.3) in R. Differentially expressed genes (DEGs) were selected using an adjusted *P*-value < 0.05. Common DEGs in prostate and head and neck cancer cell lines were identified if they exhibited the same direction of dysregulation across all cell lines.

The common upregulated DEGs across all cell lines were analysed for their involvement in biological processes using GO pathways through over-representation analysis [13] that was implemented in *R* package – genekitr (v1.2.5). The minimum gene set size was set to 20, and a false discovery rate of <0.1 was used as the cutoff to filter pathways. All filtered pathways underwent a simplification process to reduce overlap between GO terms, using the ‘Lin’ simplification method within the simGO function. The rich factor was quantified as the ratio of the number of selected genes enriched in a specific GO term to the total number of genes listed in that term. All the GO pathways were curated using the biomaRt (v2.54.1) [33] package in *R*.

### Western blot

Proteins were extracted with RIPA lysis buffer (Thermo Scientific, MA) containing 1% Protease/Phosphatase Inhibitor Cocktail (Cell Signaling Technology, MA). The supernatant was obtained by centrifugation at maximum speed for 10 mins at 4 °C. Equal amounts of protein were loaded onto a 4–15% Mini-PROTEAN™ TGX Stain-Free™ Protein Gel (Bio-Rad, CA) and blotted onto 0.45 µm low fluorescence PVDF membrane (Bio-Rad, CA). The membrane was blocked with EveryBlot Blocking Buffer (Bio-Rad, CA) for 10 mins and incubated overnight at 4 °C with the following primary antibodies: anti-p-53BP1 (1:1000, #2675, Cell Signaling Technology [CST], MA); p-H2AX (1:1000, #80312, CST); anti-p-DNA-PKcs (1:1000, #68716, CST); RAD51 (1:1000, #8875, CST); MSH2 (1:1000, #2017, CST); p-p53 (1:1000, #82530, CST); p21 (1:1000, #2947, CST); H3K9me3 (1:1000, #13969, CST); H3K27me3 (1:1000, #9733, CST), GAPDH (1:3000, #5174, CST) was used as a housekeeping protein. PVDF membranes were subsequently incubated with HRP-linked secondary antibodies, diluted in 1:2000 with 1X TBST for 1 h. Signals were detected by incubating with SuperSignal™ West Pico PLUS Chemiluminescent Substrate (Thermo Scientific, MA), prepared according to the manufacturer’s instructions. Blots were imaged using ChemiDoc Imaging System (Bio-Rad, CA). The original Western blots are shown in the Supplementary file.

### Immunofluorescence assay

Cells were cultured on glass coverslips in 6-well plates 24 h before IR. At 1 h post-IR, the cells were fixed with 4% paraformaldehyde for 10 min, washed with cold 1X PBS, and blocked with blocking buffer (5% BSA, 1X PBS, and 0.3% Triton-X100). Cells were incubated with the following primary antibodies overnight at 4 °C: γH2AX (1:400, #3174, CST); anti-p-53BP1 (1:600, #2675, CST); H3K9me3 (1:1000, #13969, CST); H3K27me3 (1:2000, #9733, CST). The cells were then washed with 1X PBS and subsequently incubated with secondary antibodies for 1 h: Alexa Fluor® 488 Conjugate (1:1000, #4408, CST); Alexa Fluor® 647 Conjugate (1:1000, #4414, CST). Following that, cells were incubated with DAPI diluted in 1:2000 1X PBS for 8 mins and mounted in ProLong™ Gold Antifade Mountant (Invitrogen, MA), then imaged with a Confocal Microscope (Leica TCS SP8, Germany) at 100x magnification.

### Immunofluorescence foci counting and intensity analysis

Co-localised foci (γH2AX and p-53BP1) and H3K27me3 foci were quantified using Imaris software (version 9.0.1, Oxford Instruments, UK). The mean number of foci per cell was scored with a minimum of 50 cells per sample from individual triplicates. H3K9me3 intensities were analysed using open-source Fiji software (National Institutes of Health, Maryland). Nucleus regions of interest were segmented using DAPI staining. The frequency percentage of MGV per cell was used to generate the probability density for each time point and a minimum of 80 cells with intensities ≥40 was scored for triplicates. The probability density function was generated based on kernel density estimation in *R* v4.1.2. The total probability for cells with intensities ≥40 was computed and the area under the curve was plotted.

### Real-time qPCR

1 µg of RNA was converted to cDNA using the iScript™ Reverse Transcription Supermix. Next, RT-qPCR (Bio-Rad, CA) was performed using iTaq Universal SYBR Green Supermix (Bio-Rad, CA). The reactions were performed under the following conditions: Preheating at 90 °C for 30 s, followed by 39 cycles of denaturation at 95 °C for 5 s, and annealing at 60 °C for 30 s. Fold changes in gene expression were calculated using the *ΔΔ*CT method, *n* = 3 per group.

The following primer sequences were used in this study:

BAHD1 - FWD: 5’ AGATCTCTGCCCTCTGGGAG 3’

BAHD1 - REV: 5’ TTCATTCTGCAAGGGCTCGT 3’

GAPDH - FWD: 5’ ACTAGGCGCTCACTGTTCT 3’

GAPDH - REV: 5’ GACCAAATCCGTTGACTCCG 3’

### siRNA transfection of BAHD1

Cells were cultured in a 60 mm dish and treated with siBAHD1 (10 nM) solution containing Opti-MEM™ I Reduced Serum Medium (Gibco, New York) and Lipofectamine™ RNAiMAX Transfection Reagent (Invitrogen, MA) for 48 h. The small interfering RNA (siRNA) used in this study were purchased from (Integrated DNA Technologies, Lowa). The knockdown of BAHD1 was then performed using the TriFECTa® RNAi Kit (Integrated DNA Technologies, Lowa), and scrambled negative control was used in this study.

The siRNA sequences used in this study are indicated below:

BAHD1 – sense: 5’GACAGAGAUAAGAAGUACUUCUUTA 3’

BAHD1 – anti-sense: 5’ UAAAGAAGUACUUCUAAUCUCUGUCAA 3’

### National Cancer Centre Singapore (NCCS) cohort and gene expression profiling

The NCCS prostate cancer cohort comprised 151 patients (Supplementary Table 3) diagnosed with biopsy-proven localised prostate adenocarcinoma who underwent treatment with radiotherapy with or without androgen deprivation therapy from April 21, 2011 to June 17, 2022. Treatment details, tumour sampling, and gene expression profiling were as previously described [17] by using the Decipher genomic classifier platform (Veracyte Inc, San Francisco, CA, USA).

The NCCS head and neck cancer cohort comprised 158 patients (Supplementary Table 4) who were newly diagnosed with histologically proven nasopharyngeal carcinoma between November 1987 and February 2024. These patients were treated with either intensity-modulated radiation therapy (IMRT) alone or concurrent chemoradiotherapy (CCRT).

RNAseq was used to generate the gene expression data of the NCCS head and neck cancer cohort. Stranded RNAseq libraries were prepared using the TruSeq RNA Exome kit (Illumina, CA). 150 bp paired-end sequencing was performed using Novaseq 6000 (Illumina, CA) with at least 50 million reads per sample. Both library preparation and sequencing were carried out by NovogeneAIT Genomics Singapore Pte Ltd. Adapter sequences and low-quality base calls were removed from the raw sequence reads using Trim Galore (v0.6.4) and Cutadapt (v2.10). Trimmed reads were then mapped to the hg38 human reference genome using Spliced Transcripts Alignment to a Reference (STAR v2.6.1 d) with standard settings. Gene quantification was performed with the “--quantMode GeneCounts” option in STAR.

### External cohort for prostate and head and neck cancers

An independent prostate cancer cohort from our ongoing collaboration with Genomic research discovery database (GRID) was used, as described in a published study [18]. This cohort consists of 121 patients diagnosed with prostate cancer and treated with radiotherapy without ADT (Supplementary Table 5). Gene expression profiling was conducted using the same microarray platform as the NCCS prostate cancer cohort, as previously described [17].

For the external head and neck cancer cohort, a publicly available dataset consisting of 113 treatment-naïve, primary undifferentiated nasopharyngeal carcinoma samples (Supplementary Table 6) from a published study [19]. The processed RNAseq normalised counts matrix was downloaded from the Gene Expression Omnibus under accession number GSE102349. The original study utilized Omicsoft ArraySuite software to process their RNAseq data.

### Calculation of heterochromatin formation enrichment score

The enrichment scores for the heterochromatin formation pathway were calculated for each patient using single sample gene set enrichment analysis (ssGSEA) [34]. The gene set for the heterochromatin formation pathway (GO: 0031507) was curated using the biomaRt (v2.54.1) [35] package in *R*, and a total of 91 genes were included in this gene set. To compute the enrichment scores, we utilised the GSVA (v1.46.0) [35] package from *R* with the method set to ‘ssgsea’ and all other parameters set to default values as set in the function. The median and interquartile range of the enrichment scores for each cohort are listed in Supplementary Tables 3–6, respectively.

### Clinical survival endpoint

Distant metastasis-free survival (DMFS) was used as the clinical endpoint for the NCCS prostate cancer cohort and the GRID prostate cancer cohort. For the head and neck cohorts, locoregional recurrence-free survival (LRFS) was used as the clinical endpoint in the NCCS head and neck cancer cohort. It was defined as the time interval from the date of diagnosis to the date of clinical or radiological occurrence of locoregional relapse and/or death from any cause. For the external head and neck cancer cohort (GSE102349), the available clinical endpoint provided by the original study [19] was disease-free survival (DFS). It was defined as the time interval from the date of diagnosis to the date of tumour progression.

### Association of heterochromatin activity and patient survival after radiotherapy

We hypothesised that a higher of heterochromatin activity correlates with an increased risk of relapse following radiotherapy. To test this hypothesis, each cohort was stratified based on the level of heterochromatin formation (low versus high) using median, tertile, quartile, or quintile cutoffs in a stepwise approach. The log-rank *P*-value was used as an indicator to determine the optimal stratification cutoff for heterochromatin formation levels.

To this end, we defined “high” heterochromatin activity based on different cutoff points for each cohort: top 3 quintiles (>1.845) for the NCCS prostate cancer cohort, uppermost quartile (>5.088) for the NCCS head and neck cohort, the median (>0.505) for the GRID prostate cancer cohort, and uppermost tertile (>4.663) for the GSE102349 head and neck cohort.

The Kaplan-Meier method was used to generate survival curves, and the reverse Kaplan-Meier method was used to calculate the median survival follow-up time for NCCS cohorts. The hazard ratio was computed using the Cox proportional hazards regression model. *R* package survival (v3.4) was used for the survival analyses.

### Statistical analysis

Each experiment was conducted at least three times independently. GraphPad Prism 8 software (version 8.0.2 [263]) and *R* v4.1.2 were used for statistical analysis and data visualisation. *P*-values were calculated by two-tailed Student’s *t*-test (paired) to compare the differences between the two groups. *P* < 0.05 was considered statistically significant, *P < 0.05, **P < 0.01, ***P < 0.001; NS, not significant.

## Supplementary information

Supplementary Appendix

Western Blot Original

## Data Availability

Cell line genomic, and transcriptomic raw sequencing data were deposited to SRA under the accession number PRJNA1166646. The NCCS patient data used in this study are not publicly available due to patient privacy requirements but are available upon reasonable request from the authors. Requests may be submitted via email to M.L.K.C. (gmsclkm@nus.edu.sg).
