# Supplementary material for: Genomic and transcriptomic profiling of radioresistant prostate and head and neck cancers implicate a BAHD1-dependent modification of DNA damage at the heterochromatin
Source: Cell Death Dis. 2024 Dec 24;15(12):929. doi: 10.1038/s41419-024-07316-y (PMC11668898; doi:10.1038/s41419-024-07316-y)
Supplement: Supplementary file 1 — Supplementary Appendix [file 41419_2024_7316_MOESM1_ESM.docx]

**Supplementary Appendix**

Chaw Yee Beh, Celestia Pei Xuan Yeo, Boon Hao Hong et al. *Genomic and Transcriptomic Profiling of Radioresistant Prostate and Head and Neck Cancers Implicate a BAHD1-dependent Modification of DNA Damage at the Heterochromatin*

p 1. Supplementary Tables

p 2. Table 1. Summary of COSMIC mutational signatures in prostate cancer cell lines (22Rv1, DU145) and head and neck cancer cell lines (FaDu, HK1).

p 3. Table 2. Gene ontology (GO) pathways curated for 5 upregulated genes in 4 RR cell lines by over-representation analysis (false-discovery rate <0.1).

p 4. Table 3. Summary of clinical characteristics of NCCS prostate cancer cohort.

p 5. Table 4. Summary of clinical characteristics of NCCS head and neck cancer cohort.

p 6. Table 5: Summary of clinical characteristics of Berlin et al. prostate cancer cohort from GRID.

p 7. Table 6: Summary of clinical characteristics Zhang et al. (GSE102349) head and neck cancer cohort.

p 8. Supplementary Figures

p 8. Figure S1. Volcano plot of differentially expressed genes after multiple hypothesis testing (*P* < 0.05) in prostate and, head and neck cancer cell lines.

p 9. Figure S2. Principal component analysis of 4 cell lines.

p 10. Figure S3. Gene expression of DNA repair geneset (GO:0006281) in all cell lines.

p.11. Figure S4. Evaluation of DNA damage responses of 22Rv1 and FaDu-RR cells relative to parental WT cells post-IR.

p 12. Figure S5. Protein expression of MSH6 and MLH1 in 22Rv1- and FaDu-RR cells relative to their parental WT cells.

p 13. Figure S6. The heterochromatin status of 22Rv1 and FaDu-RR cells relative to parental WT cells post-IR.

p 14. Figure S7. Representative immunofluorescence microscopy images with different intensities of H3K9me3 (green)

p 15. Figure S8. Real-time qPCR validation of BAHD1 mRNA expression levels post-siRNA treatment.

p 16. Figure S9. The heterochromatin status with and without siBAHD1 treated-22Rv1-RR cells relative to the parental WT cells.

p 17. Figure S10. The heterochromatin status with and without siBAHD1 treated-FaDu-RR cells relative to the parental WT cells.

p 18. Figure S11. Evaluation of BAHD1 knockdown on the radiosensitivity of 22Rv1-RR and FaDu-RR cells relative to their parental WT cells.

p 19. Figure S12. Investigation of the clonogenic survivability of RR cells relative to the parental WT cells.

p 20. References

***Supplementary Tables***

Table 1. **Summary of COSMIC mutational signatures in prostate and, head and neck cell lines.**

Prostate (22Rv1, DU145)

| **Signature** | **Aetiology** |
| --- | --- |
| **DNA repair related** | |
| SBS6 | Defective DNA mismatch repair |
| SBS15 | Defective DNA mismatch repair |
| SBS20 | Concurrent *POLD1* mutations and defective DNA mismatch repair |
| **Others** | |
| SBS19 | Unknown |
| SBS31 | Prior chemotherapy treatment with platinum drugs |
| SBS84 | Activity of activation-induced cytidine deaminase (AID) |

Head and neck (FaDu, HK1)

| **Signature** | **Aetiology** |
| --- | --- |
| **DNA repair related** | |
| SBS3 | Defective homologous recombination-based DNA damage repair |
| SBS14 | Concurrent polymerase epsilon mutation and defective DNA mismatch repair |
| SBS21 | DNA mismatch repair deficiency |
| **Others** | |
| SBS53 | Possible sequencing artefact |
| SBS39 | Unknown |

Table 2. **Gene ontology (GO) pathways curated for 5 upregulated genes in 4 RR cell lines by over-representation analysis (false-discovery rate <0.1).**

| **GO pathway ID** | **Description** | **Genes involved** | **Number of genes listed** |
| --- | --- | --- | --- |
| GO:0031507 | Heterochromatin formation | *BAHD1* | 91 |
| GO:0150104 | Vascular transport | *ATP8A1* | 87 |
| GO:0097035 | Membrane organisation | *ATP8A1* | 63 |
| GO:0002504 | Immune response | *CTSD* | 36 |
| GO:0060416 | Growth hormone receptor | *JAK1* | 37 |
| GO:1903670 | Sprouting angiogenesis | *JAK1* | 60 |
| GO:0034110 | Cell-cell adhesion | *JAK1* | 37 |
| GO:0007260 | STAT phosphorylation | *JAK1* | 86 |
| GO:0034381 | Lipoprotein clearance | *MYLIP* | 53 |
| GO:0043112 | Metabolic process | *MYLIP* | 61 |

Table 3. **Summary of clinical characteristics of NCCS prostate cancer cohort.**

| **Characteristics** | **N=151** |
| --- | --- |
| **Median age at diagnosis, years (IQR)** | 71.6 (67.6-75.7) |
| **Median survival follow-up time, years (IQR)** | 5.2 (5.0-5.7) |
| **NCCN 2021** [1] |  |
| Low | 3 (2.0) |
| Favourable intermediate | 23 (15.2) |
| Unfavourable intermediate | 35 (23.2) |
| High | 23 (15.2) |
| Very high | 60 (39.7) |
| Regional | 7 (4.6) |
| **Treatment** |  |
| RT only | 37 (24.5) |
| RT+short term ADT (<6 months) | 33 (21.9) |
| RT+long term ADT (≥6 months) | 81 (53.6) |
| **Median heterochromatin formation enrichment score (IQR)** | 1.895 (1.759-2.024) |
| **Heterochromatin formation enrichment level** |  |
| Low (≤1.845) | 61 |
| High (>1.845) | 90 |

Abbreviation: IQR = interquartile range; RT = radiotherapy; ADT = androgen deprivation therapy.

Table 4. **Summary of clinical characteristics of NCCS head and neck cancer cohort.**

| **Characteristics** | **N=158** |
| --- | --- |
| **Median age at diagnosis, years (IQR)** | 54.8 (46.6-61.9) |
| **Median survival follow-up time, years (IQR)** | 6.4 (4.2-8.4) |
| **Sex (%)** |  |
| Male | 120 (75.9) |
| Female | 38 (24.1) |
| **TNM-stage by AJCC/UICC 8^th^ edition** [2] |  |
| I | 10 (6.3) |
| II | 31 (19.6) |
| III | 53 (33.5) |
| IVA | 64 (40.5) |
| **Treatment** |  |
| RT only | 36 (22.8) |
| IC+RT only | 1 (0.6) |
| IC+CCRT | 28 (17.7) |
| IC+CCRT+AC | 12 (7.6) |
| CCRT | 54 (34.2) |
| CCRT+AC | 27 (17.1) |
| **Median heterochromatin formation enrichment score (IQR)** | 4.968 (4.858-5.077) |
| **Heterochromatin formation enrichment level** |  |
| Low ( ≤5.088) | 119 |
| High (>5.088) | 39 |

Abbreviation: IQR = interquartile range; RT = radiotherapy; IC = induction chemotherapy; CCRT = concurrent chemotherapy radiotherapy; AC = Adjuvant chemotherapy.

Table 5. **Summary of clinical characteristics of Berlin et al. prostate cancer cohort from GRID.**

| **Characteristics** | **N=121** |
| --- | --- |
| **Median age at diagnosis, years (IQR)** | 72.4 (68.4-75.0) |
| **Median survival follow-up time, years (IQR)** | 7.7 (0.7-11.2) |
| **NCCN subclassification** [3] |  |
| Favourable intermediate | 33 (27.3) |
| Unfavourable intermediate | 87 (71.9) |
| Unknown | 4 (3.3) |
| **Treatment** |  |
| RT only | 121 (100) |
| **Median heterochromatin formation enrichment score (IQR)** | 0.505 (0.477-0.532) |
| **Heterochromatin formation enrichment level** |  |
| Low (≤0.505) | 71 |
| High (>0.505) | 50 |

Abbreviation: IQR = interquartile range; RT = radiotherapy.

Table 6. **Summary of clinical characteristics Zhang *et al.* (GSE102349) head and neck cancer cohort.**

| **Characteristics** | **N=88** |
| --- | --- |
| **Median survival follow-up time, years (IQR)** | 2.27 (1.64-2.97) |
| **Clinical stage by AJCC/UICC 7^th^ edition** [4] |  |
| I | 5 (5.7) |
| II | 2 (2.3) |
| III | 39 (44.3) |
| IVA | 22 (25.0) |
| Unknown | 20 (22.7) |
| **Median heterochromatin formation enrichment score (IQR)** | 4.572 (4.440-4.707) |
| **Heterochromatin formation enrichment level** |  |
| Low (≤4.663) | 59 |
| High (>4.663) | 29 |

Abbreviation: IQR = interquartile range.

***Supplementary Figures***


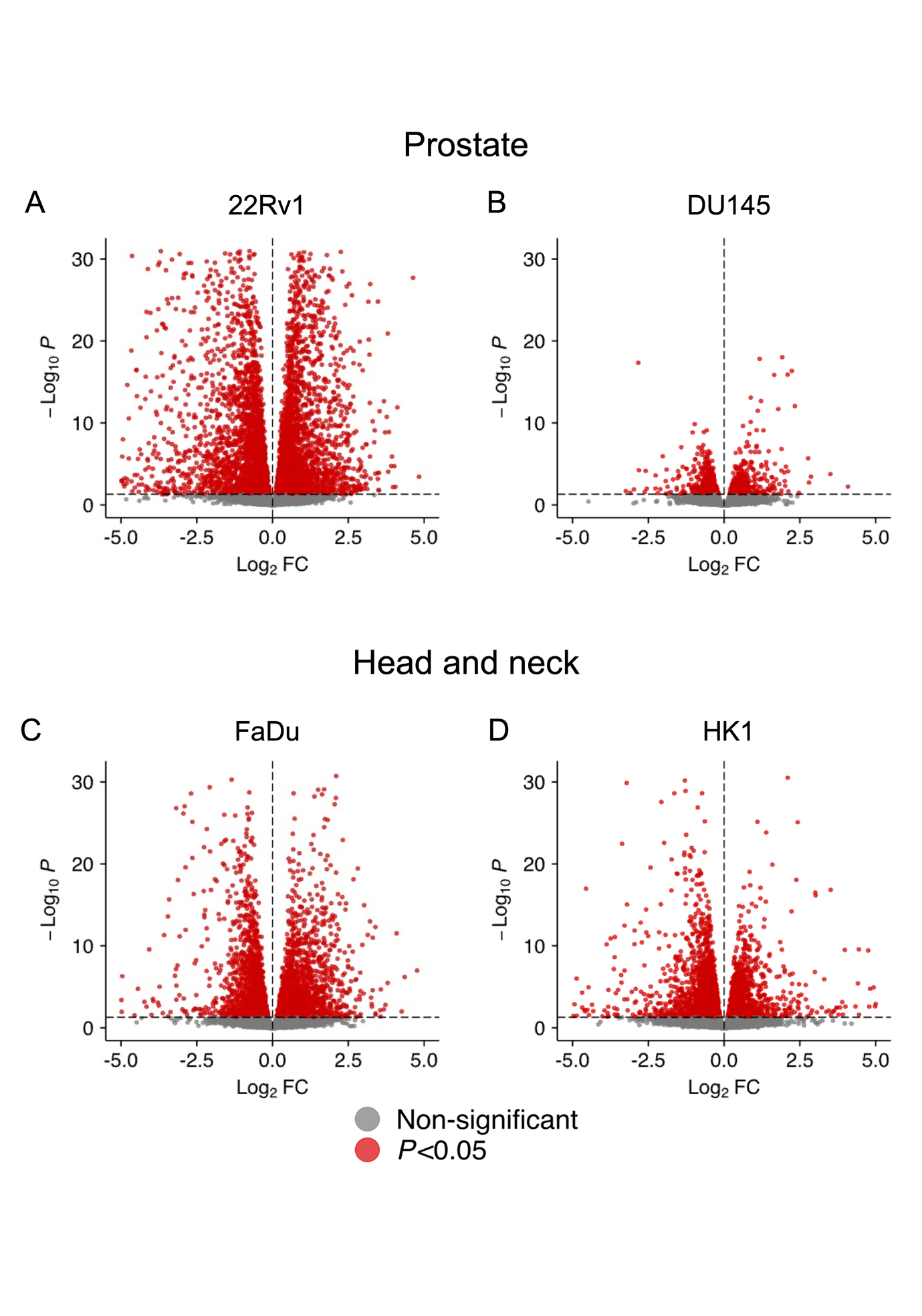


Figure S1. **Volcano plot of differentially expressed genes after multiple hypothesis testing (*P* <0.05) in prostate and, head and neck cancer cell lines. (A)** 22Rv1, **(B)** DU145; **(C)** FaDu **(D)** HK1.


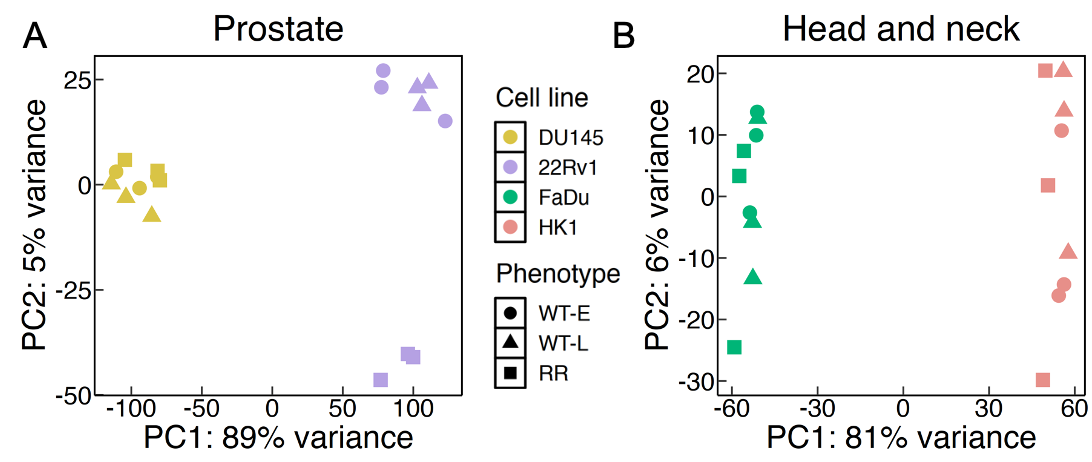


Figure S2. **Principal component analysis of 4 cell lines. (A)** prostate cell lines – 22Rv1 and DU145 **(B)** head and neck cell lines – FaDu and HK1. The cell line types are represented by colour while the shape indicates the cell phenotypes.


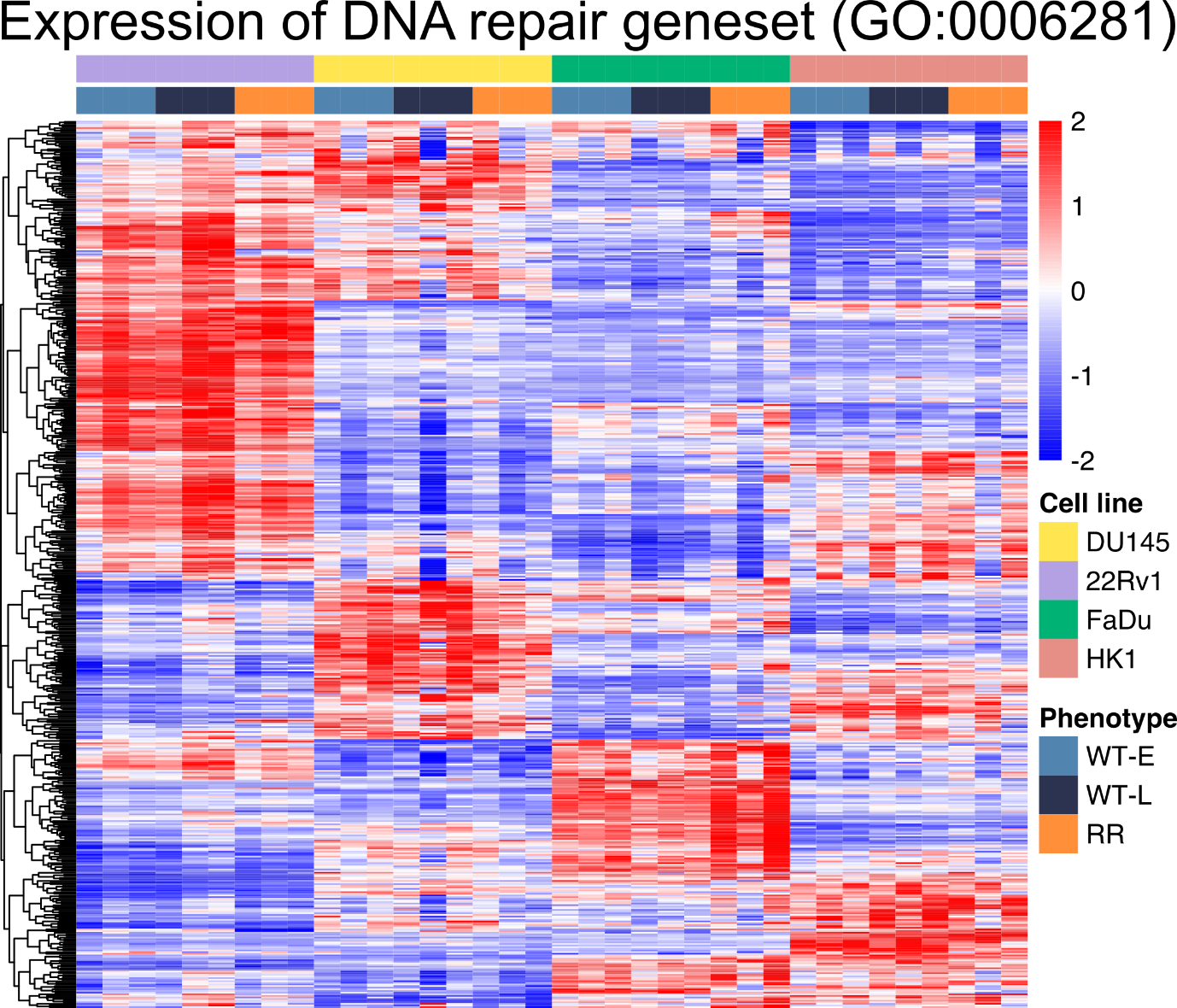


Figure S3. **Gene expression of DNA repair geneset (GO:0006281) in four cell lines.**


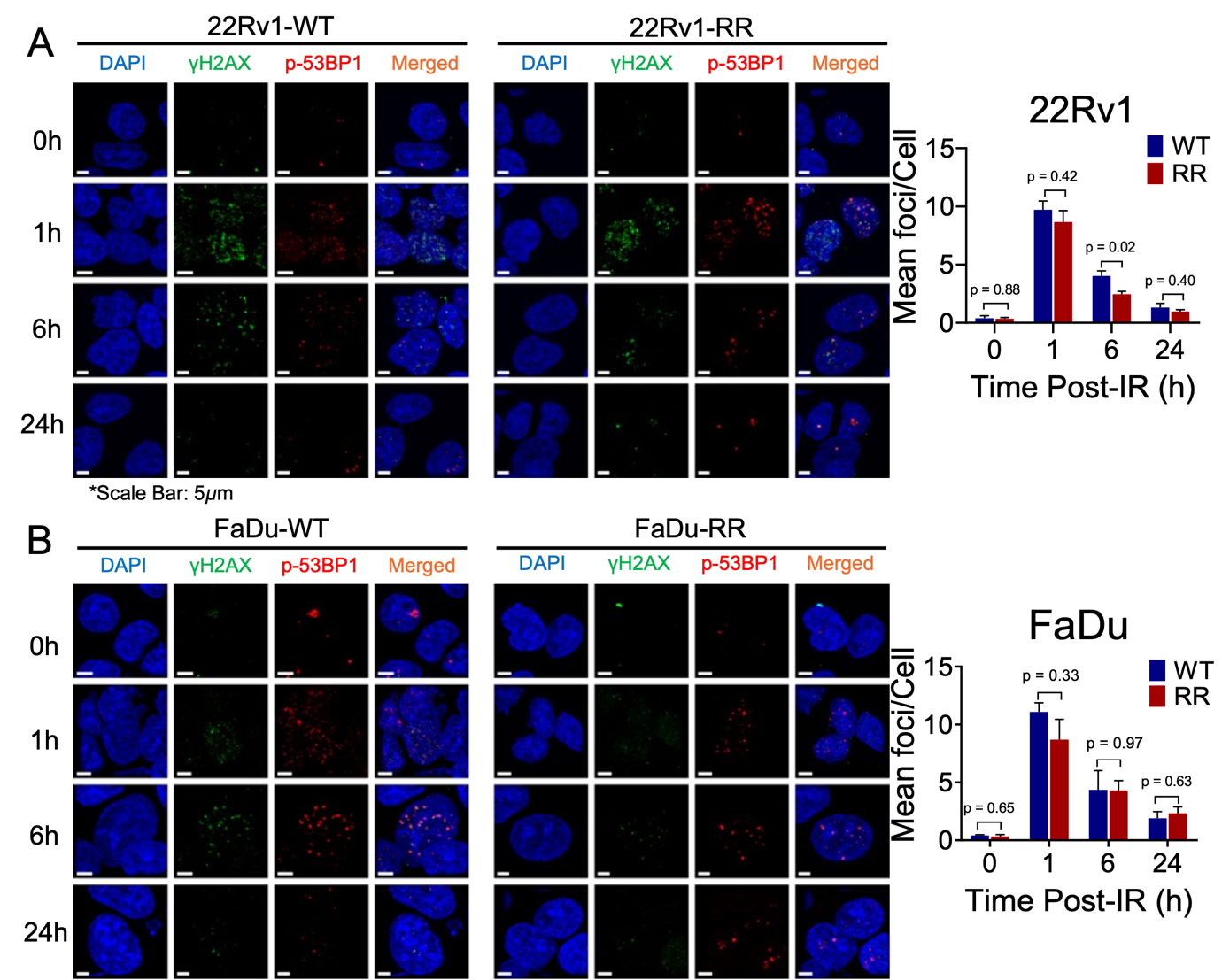


Figure S4. **Evaluation of DNA damage responses of 22Rv1 and FaDu-RR cells relative to parental WT cells post-IR.** (left) Representative images of co-localised of γH2AX (green) and p-53BP1 (red) foci at 0, 1, 6 and 24 h post-4Gy IR in (A) 22Rv1 and (B) FaDu-WT and -RR cells. DNA was counterstained with DAPI (blue). Scale bar: 5 μm. (right) Quantification of co-localised γH2AX and p-53BP1 mean foci per cell, bars represent mean±SD, n=3 per group.


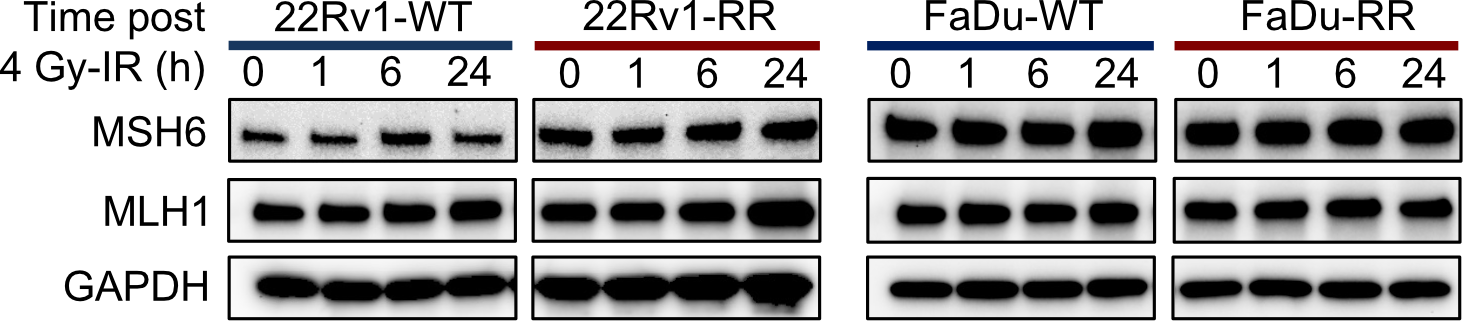


Figure S5. **Protein expression of MSH6 and MLH1 in 22Rv1- and FaDu-RR cells relative to their parental WT cells.** Representative western blot showed the changes in expression at different time points post-4 Gy (normalised against WT control), GAPDH was used as a loading control.


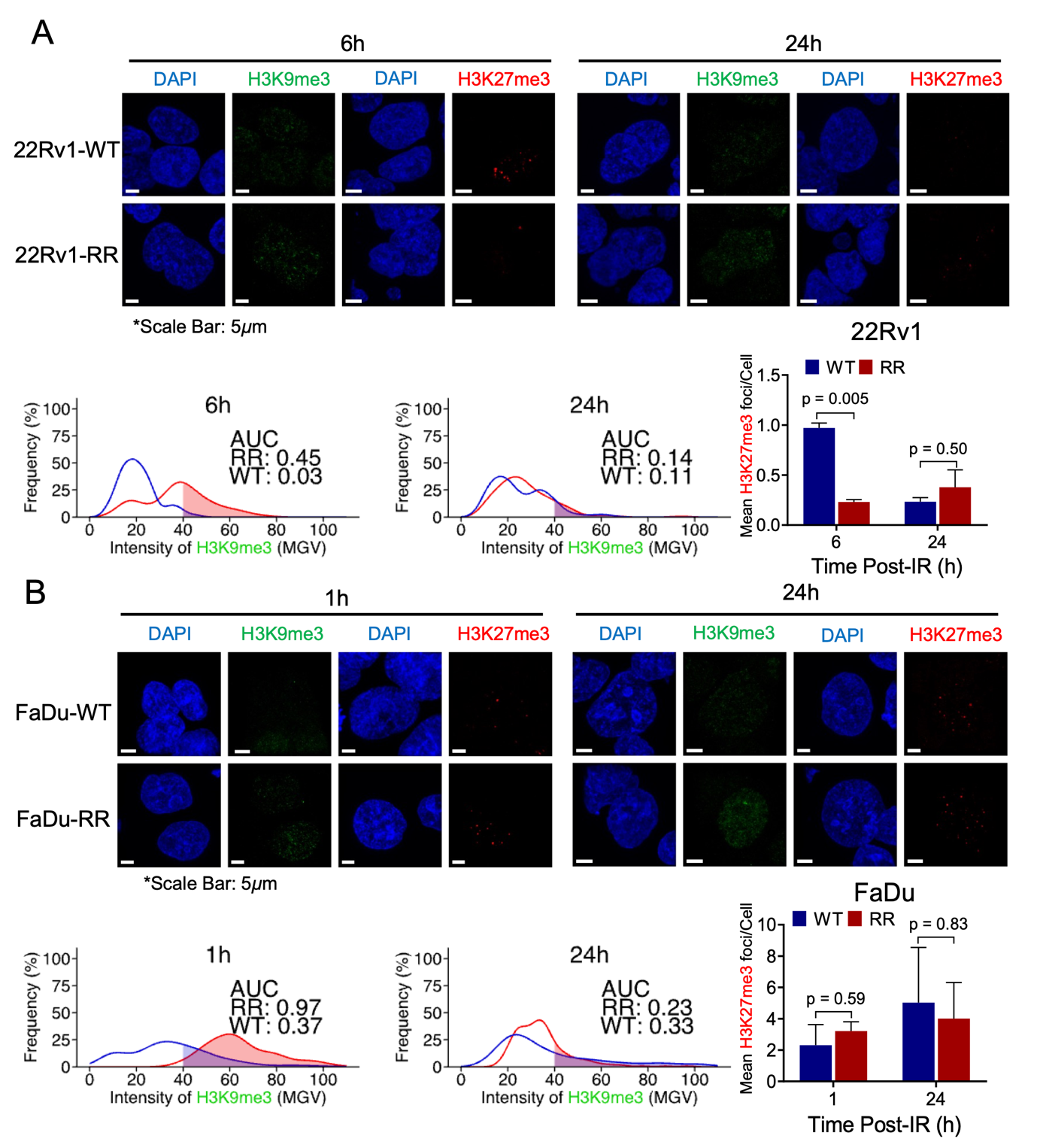


Figure S6. **The heterochromatin status of 22Rv1 and FaDu-RR cells relative to parental WT cells post-IR.** **(A and B-top panel)** Representative images of H3K9me3 (green) and H3K27me3 (red foci) in 22Rv1-WT and -RR cells at 6 and 24 h, and FaDu-WT and -RR cells at 1 and 24 h post-4Gy IR. DNA was counterstained with DAPI (blue). Scale bar: 5 μm. (bottom left) H3K9me3 intensities were quantified and represented as percentage frequencies, the AUCs were measured to compare the RR and WT; (bottom right) H3K27me3 foci were quantified as mean foci per cell, bars represent mean±SD, n=3 per group.


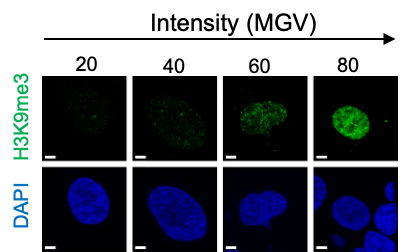


Figure S7. **Representative immunofluorescence microscopy images with different intensities of H3K9me3 (green).** Intensities values ranging from 20, 40, 60 and 80 mean gray values (MGV). DNA was counterstained with DAPI (blue).


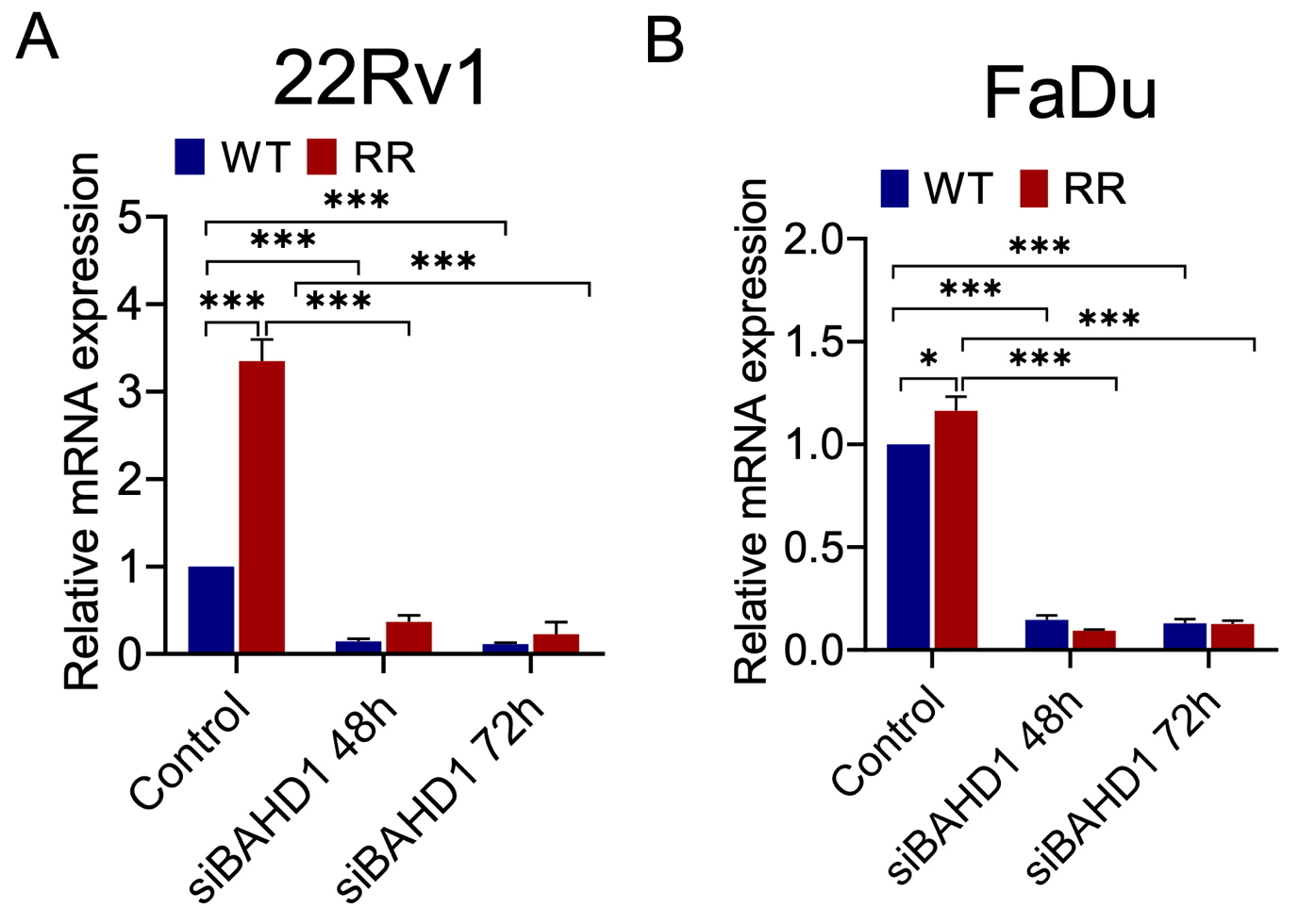


Figure S8. **Real-time qPCR validation of BAHD1 mRNA expression levels post-siRNA treatment.** Relative expression level of BAHD1 in **(A)** 22Rv1 and **(B)** FaDu-WT and -RR control, post-48 h and -72 h of siRNA treatment. Gene expression levels were normalised to their respective untreated WT control. PCR reactions for each sample were carried out in triplicates, bars represent mean±SD, n=3 per group. * Indicates significance between siBAHD1-treated and control groups. **P* <0.05, ***P* <0.01, ****P* <0.001.


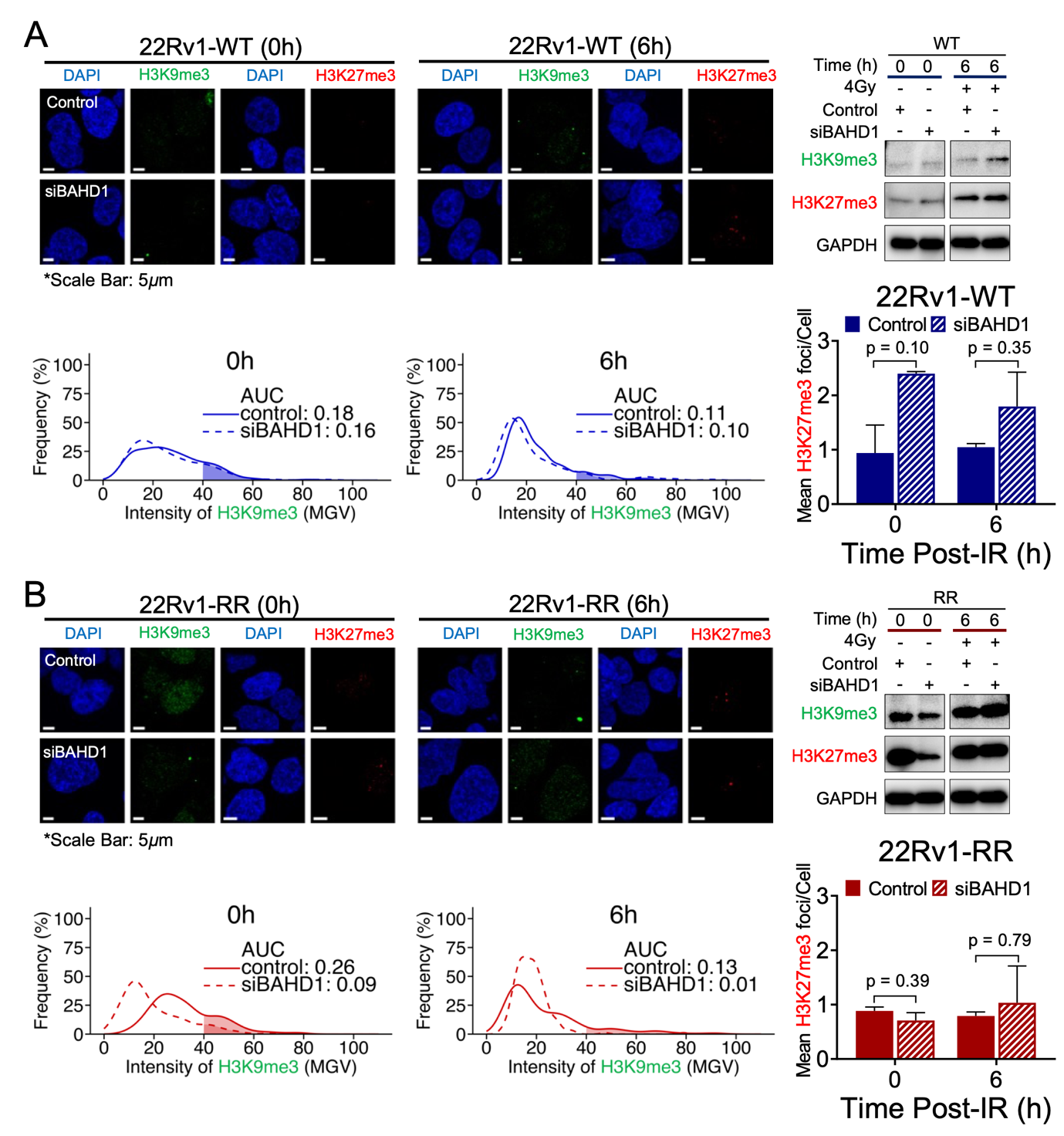


Figure S9. **The heterochromatin status with and without siBAHD1 treated-22Rv1-RR cells relative to the parental WT cells.** **(A and B-top left)** Representative images of H3K9me3 (green) and H3K27me3 (red) foci in 22Rv1-WT and 22Rv1-RR cells at 0 and 6 h treatment time points. Scale bar: 5 μm; (top right) Representative western blot showed the changes in H3K9me3 and H3K27me3 protein levels at 0 and 6 h in 22Rv1-WT and -RR cells ; (bottom left) H3K9me3 intensities were quantified and represented as percentage frequencies, the AUCs were measured to compare the RR and WT; (bottom right) H3K27me3 foci were quantified as mean foci per cell, bars represent mean±SD, n=3 per group.


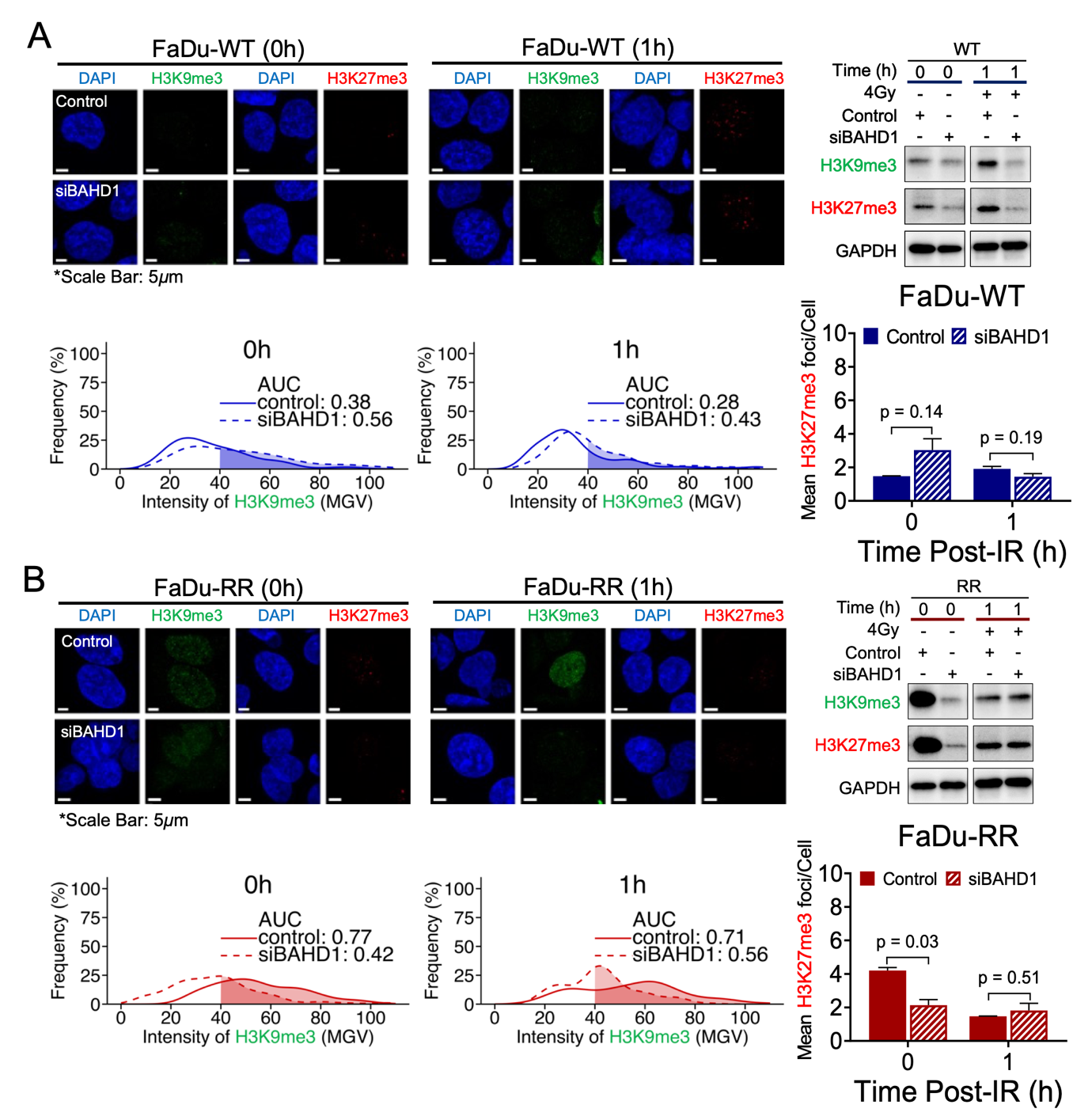


Figure S10. **The heterochromatin status with and without siBAHD1 treated-FaDu-RR cells relative to the parental WT cells.** **(A and B-top left)** Representative images of immunostaining of H3K9me3 (green) and H3K27me3 (red) foci in FaDu-WT and FaDu-RR cells at 0 and 1 h treatment time points. Scale bar: 5 μm; (top right) Representative western blot showed the changes in H3K9me3 and H3K27me3 protein levels at 0 and 1 h in FaDu-WT and -RR cells; (bottom left) H3K9me3 intensities were quantified and represented as percentage frequencies, the AUCs were measured to compare the RR and WT; (bottom right) H3K27me3 foci were quantified as mean foci per cell, bars represent mean±SD, n=3 per group.


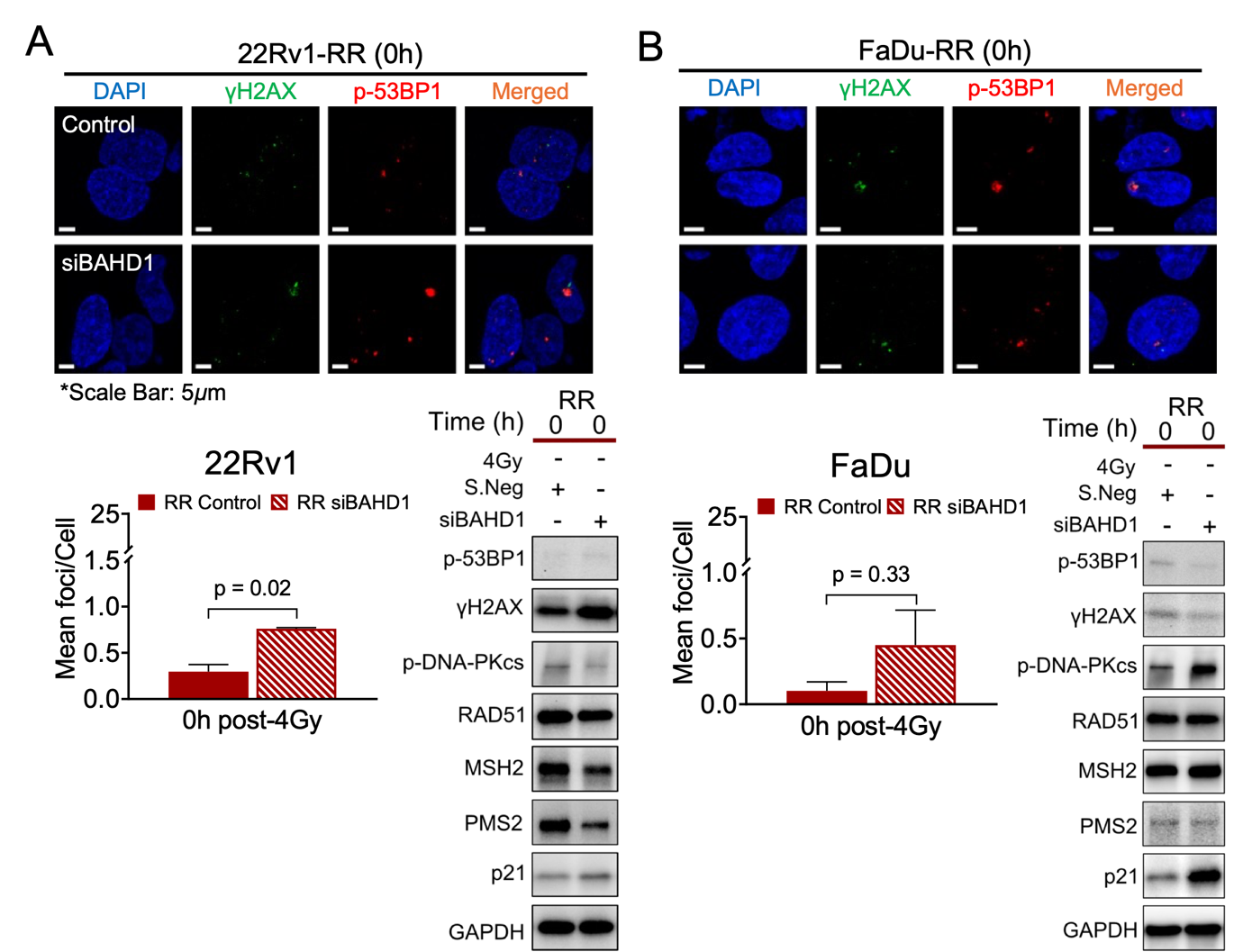


Figure S11. **Evaluation of BAHD1 knockdown on the radiosensitivity of 22Rv1-RR and FaDu-RR cells relative to their parental WT cells.** **(A and B-top)** Representative images of co-localised γH2AX (green) and p-53BP1 (red) foci in 22Rv1-RR and FaDu-RR cells at 0 h post-siBAHD1 treatment. Scale bar: 5 μm; (bottom left) Quantification of co-localised γH2AX and p-53BP1 mean foci per cell, bars represent mean±SD, n=3 per group. (bottom right) The representative western blot shows the changes in the expression of DSB, DNA repair and heterochromatin markers at 0 h post-4 Gy IR, normalised against control. GAPDH was used as a loading control.


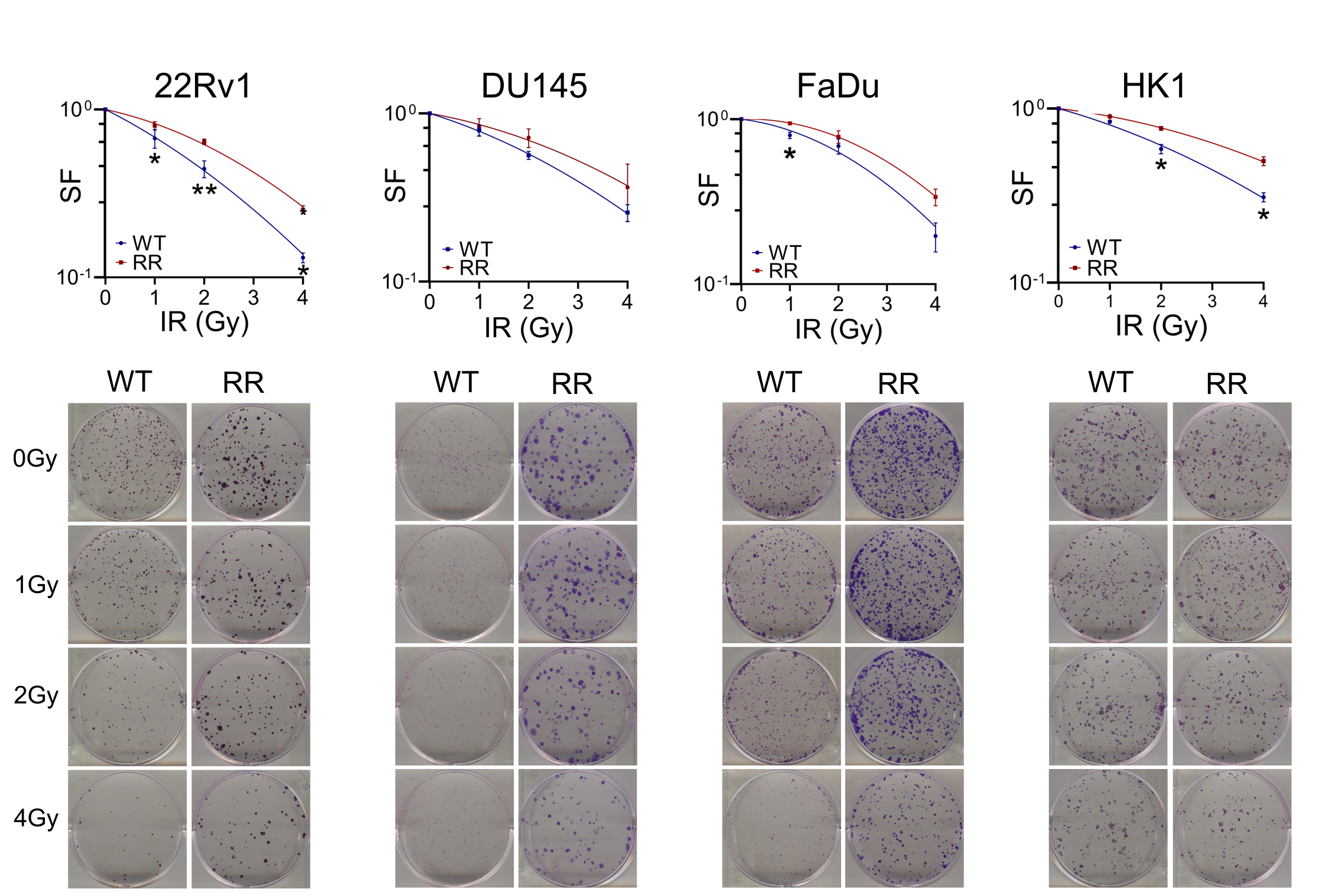


Figure S12. **Investigation of the clonogenic survivability of RR cells relative to the parental WT cells.** Colony forming assay of 22Rv1, FaDu, DU145 and HK1-WT and -RR cells post-0, 1, 2, 4 Gy irradiation. Quantification of clonogenic assays as surviving fraction relative to non-irradiated controls. Mean±SD, experiments in biological triplicate for each cell line. Two-tailed Student’s t-test between WT and RR cell lines * indicates significance between WT and RR groups. **P* <0.05, ***P* <0.01.

***References***

1. Schaeffer E, Srinivas S, Antonarakis ES, Armstrong AJ, Bekelman JE, Cheng H, et al. NCCN Guidelines Insights: Prostate Cancer, Version 1.2021. J Natl Compr Canc Netw. 2021;19:134-143.
2. Amin MB, Greene FL, Edge SB, Compton CC, Gershenwald JE, Brookland RK, et al. The Eighth Edition AJCC Cancer Staging Manual: Continuing to build a bridge from a population-based to a more "personalized" approach to cancer staging. *CA Cancer J Clin*. 2017;67:93-99.
3. Zumsteg ZS, Spratt DE, Pei I, Zhang Z, Yamada Y, Kollmeier M, et al. A new risk classification system for therapeutic decision making with intermediate-risk prostate cancer patients undergoing dose-escalated external-beam radiation therapy. *Eur Urol*. 2013;64:895-902.
4. Edge, S. B., & Compton, C. C. (2010). The American Joint Committee on Cancer: the 7th edition of the AJCC cancer staging manual and the future of TNM. *Annals of surgical oncology*, *17*, 1471–1474.
