# Supplementary material for: Genomic and transcriptomic profiling of radioresistant prostate and head and neck cancers implicate a BAHD1-dependent modification of DNA damage at the heterochromatin
Source: Cell Death Dis. 2024 Dec 24;15(12):929. doi: 10.1038/s41419-024-07316-y (PMC11668898; doi:10.1038/s41419-024-07316-y)

Figure 3 (22Rv1)

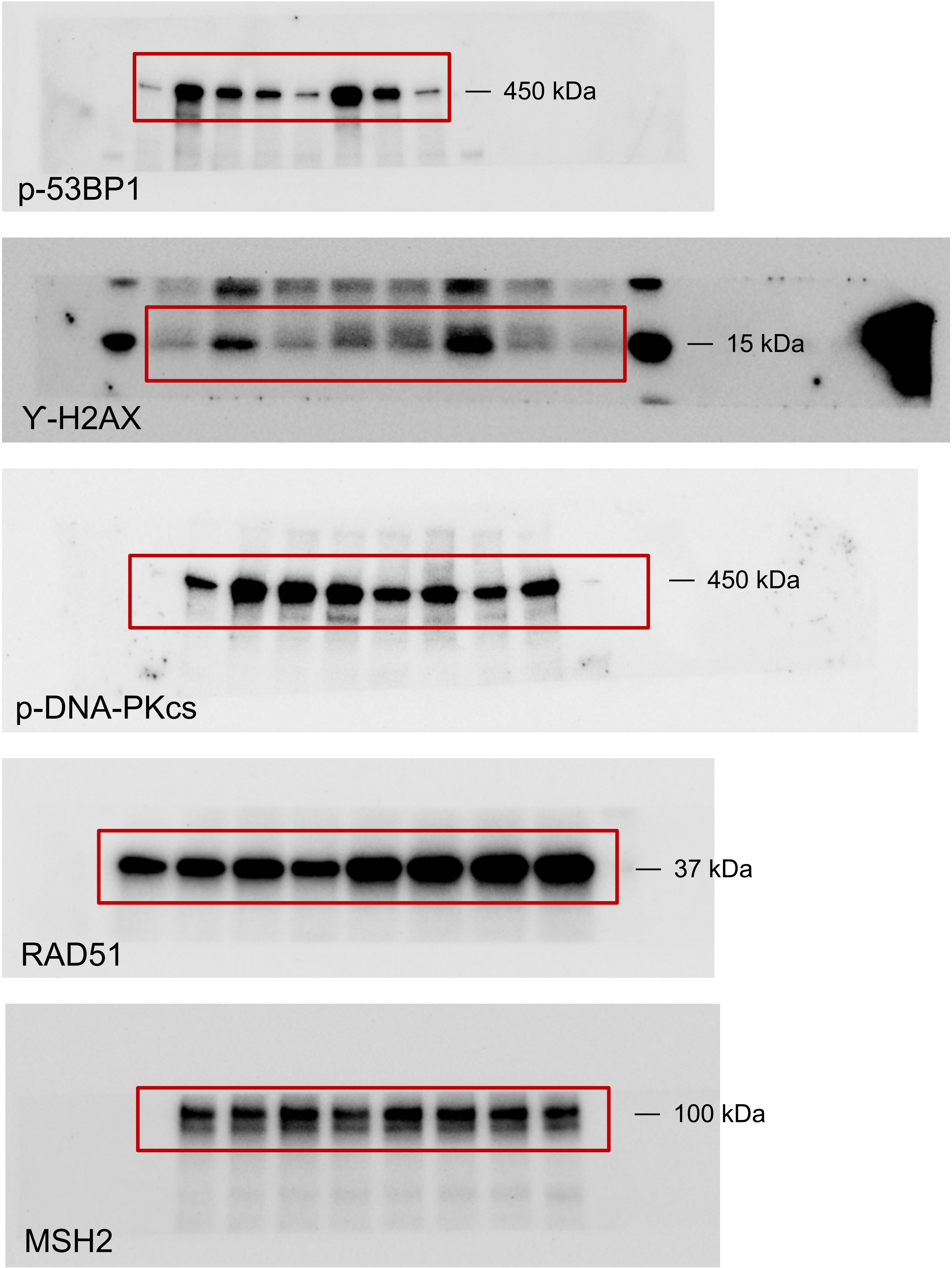

Figure 3 (22Rv1)

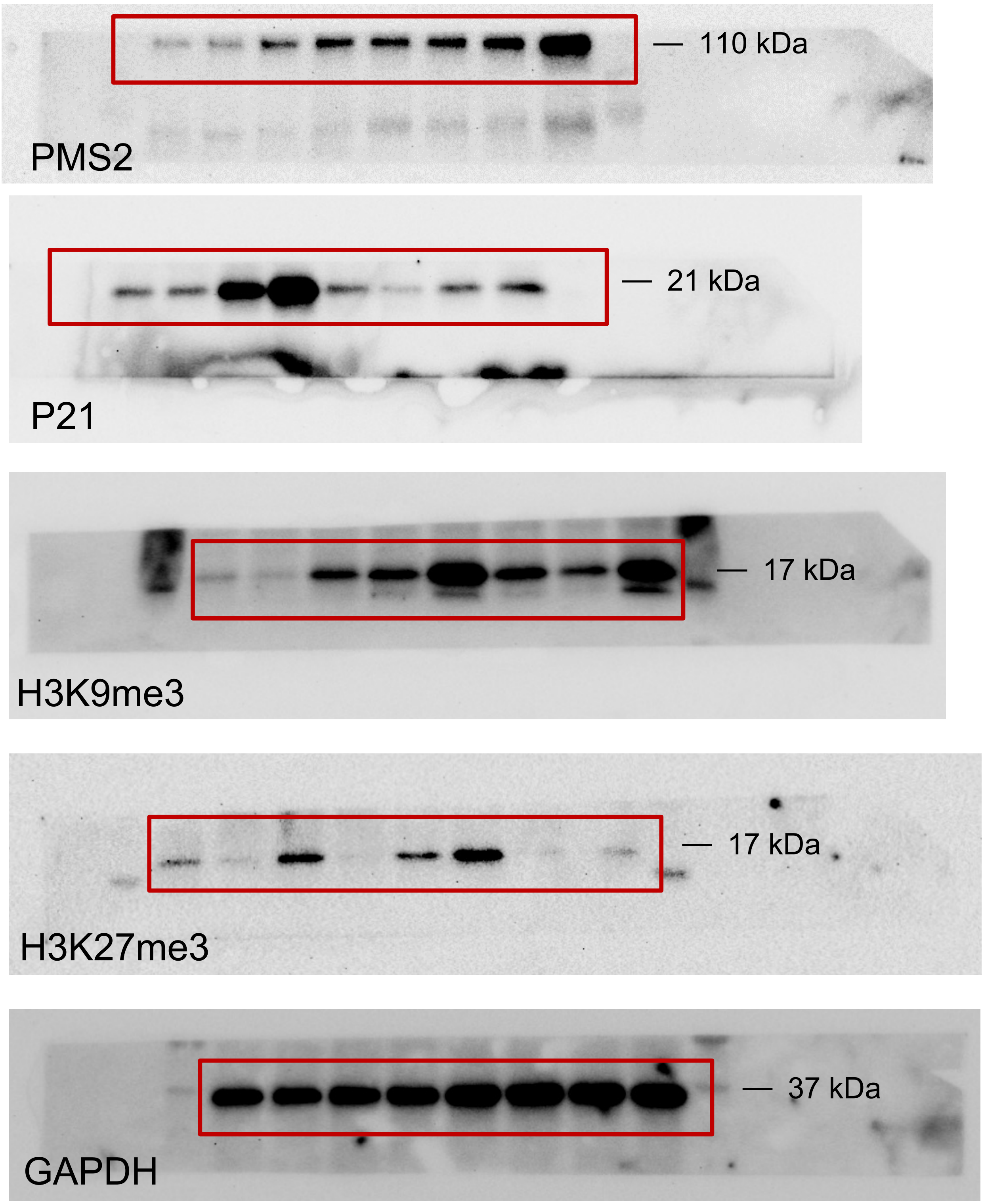

Figure 3 (FaDu)

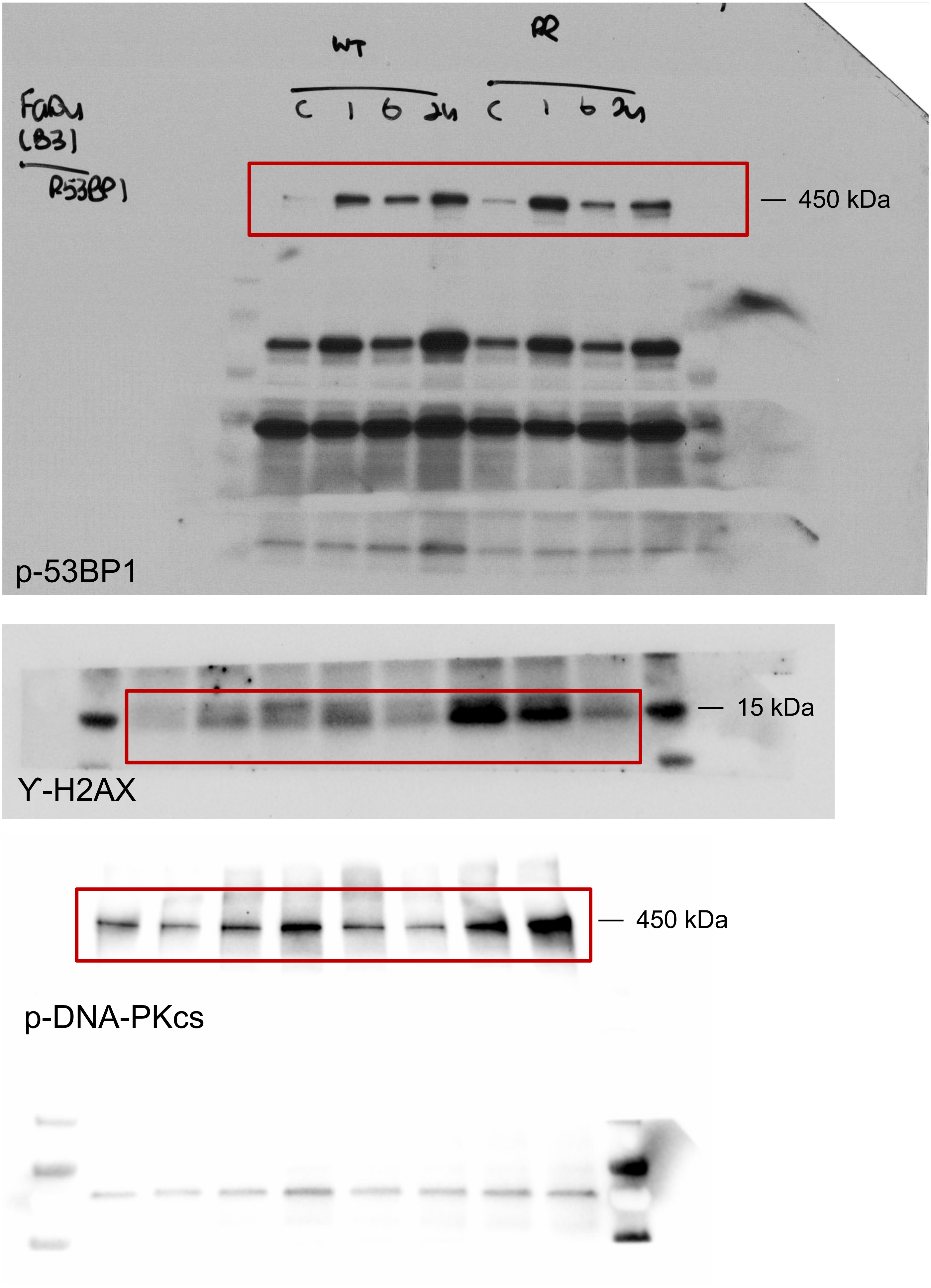

Figure 3 (FaDu)

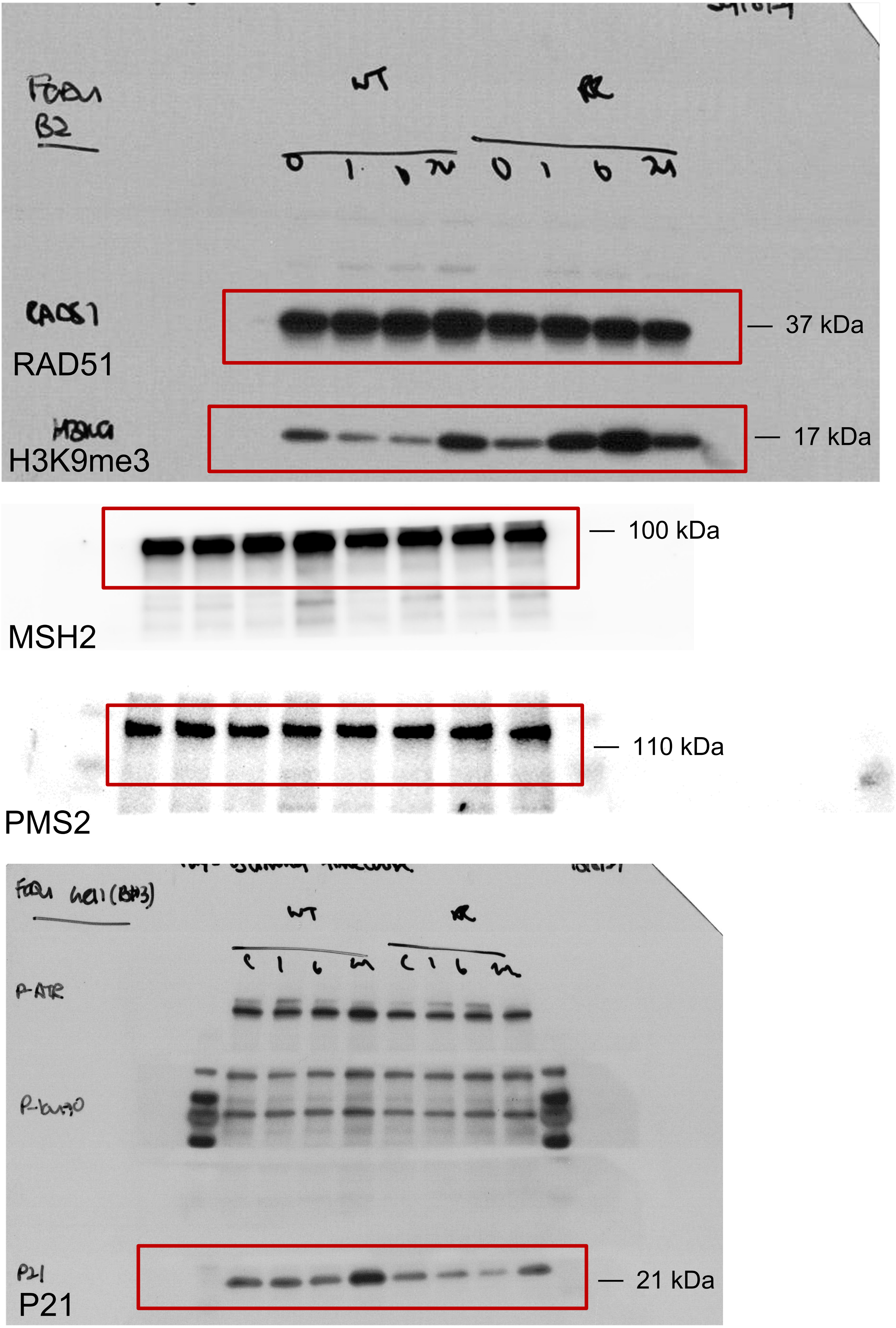

Figure 3 (FaDu)

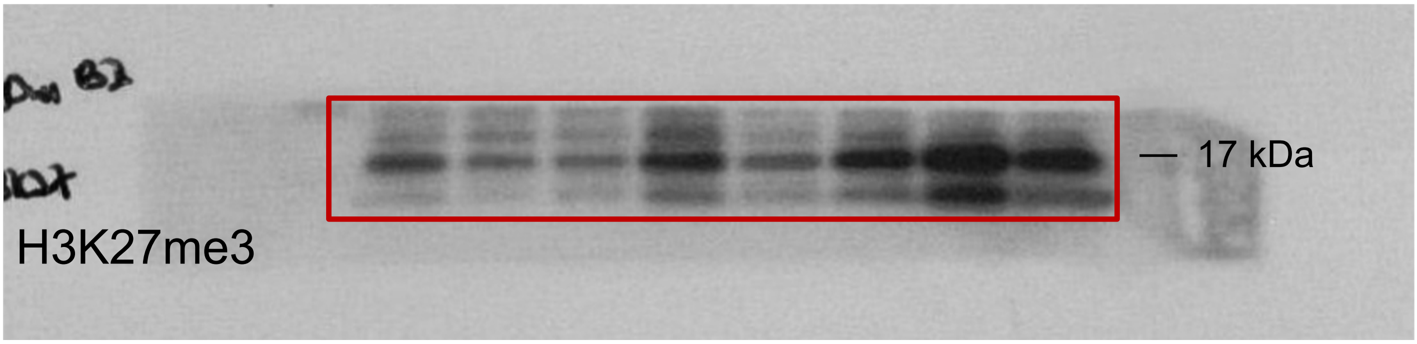

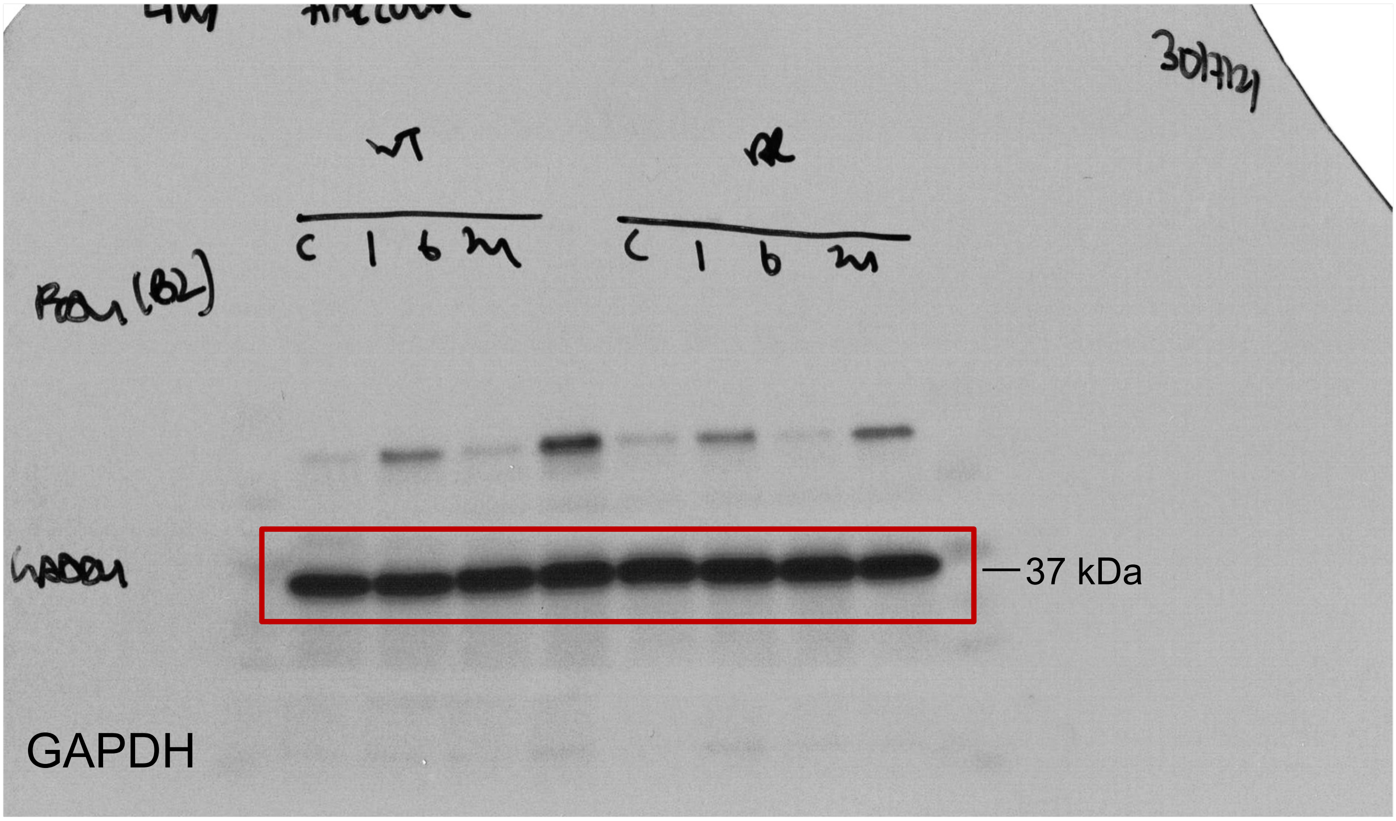

Supplementary Figure S4 (22Rv1 and FaDu)

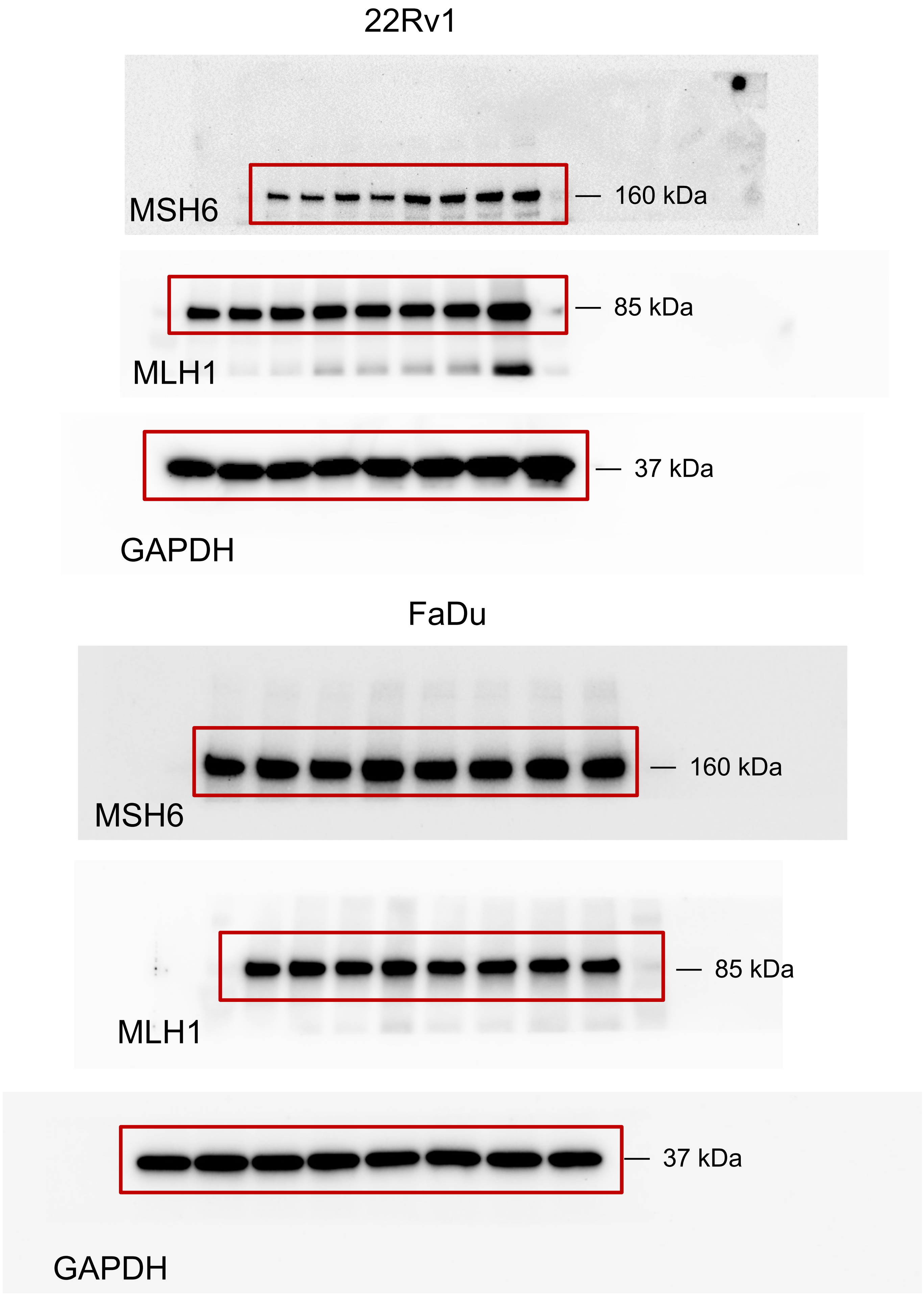

Figure 4, Supplementary Figure S7 and S8 (22Rv1 and FaDu)

22Rv1

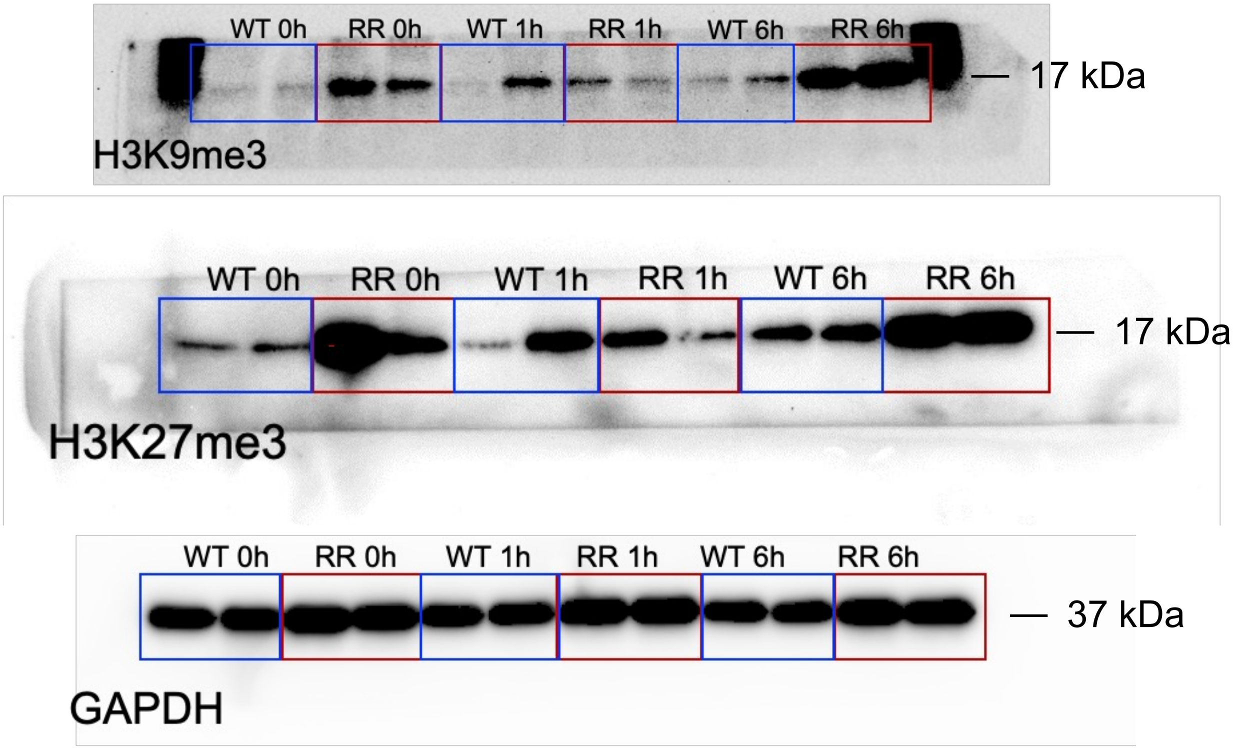

FaDu

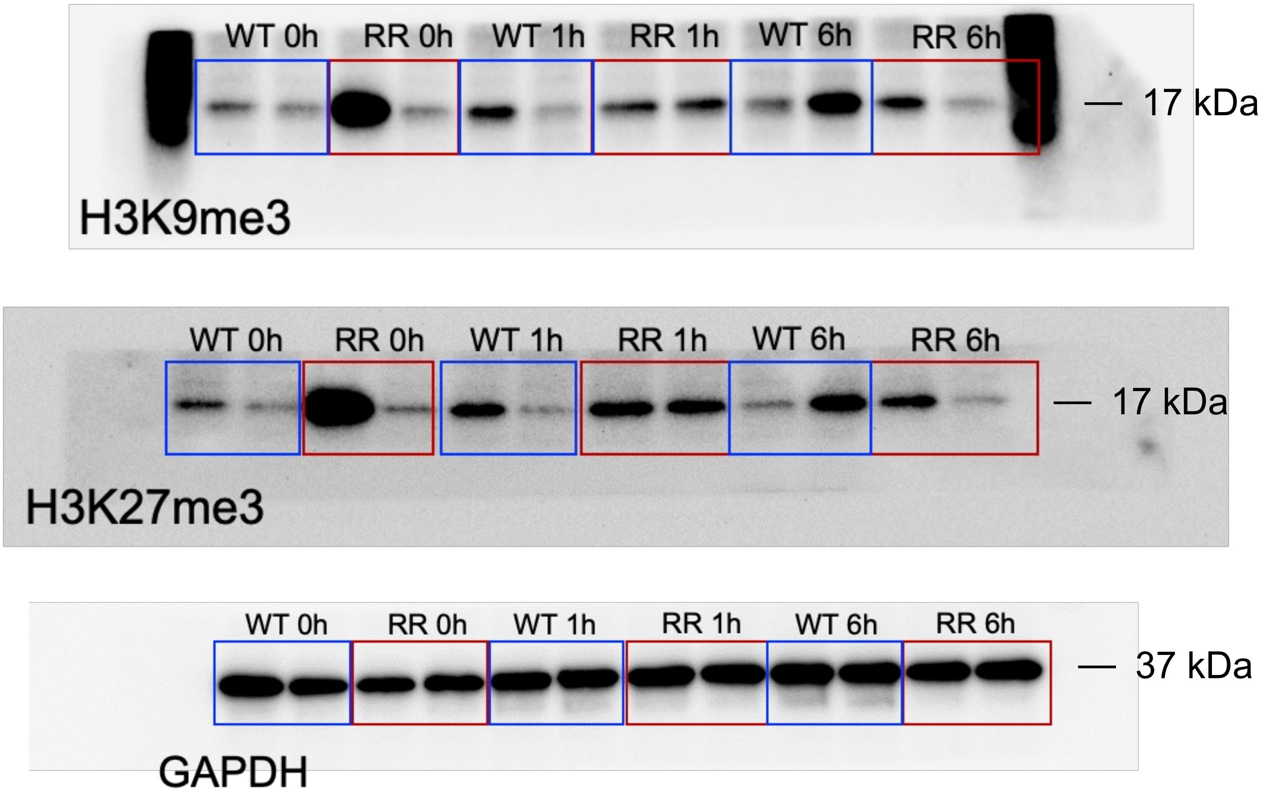

Figure 5 and Supplementary Figure S9 (22Rv1)

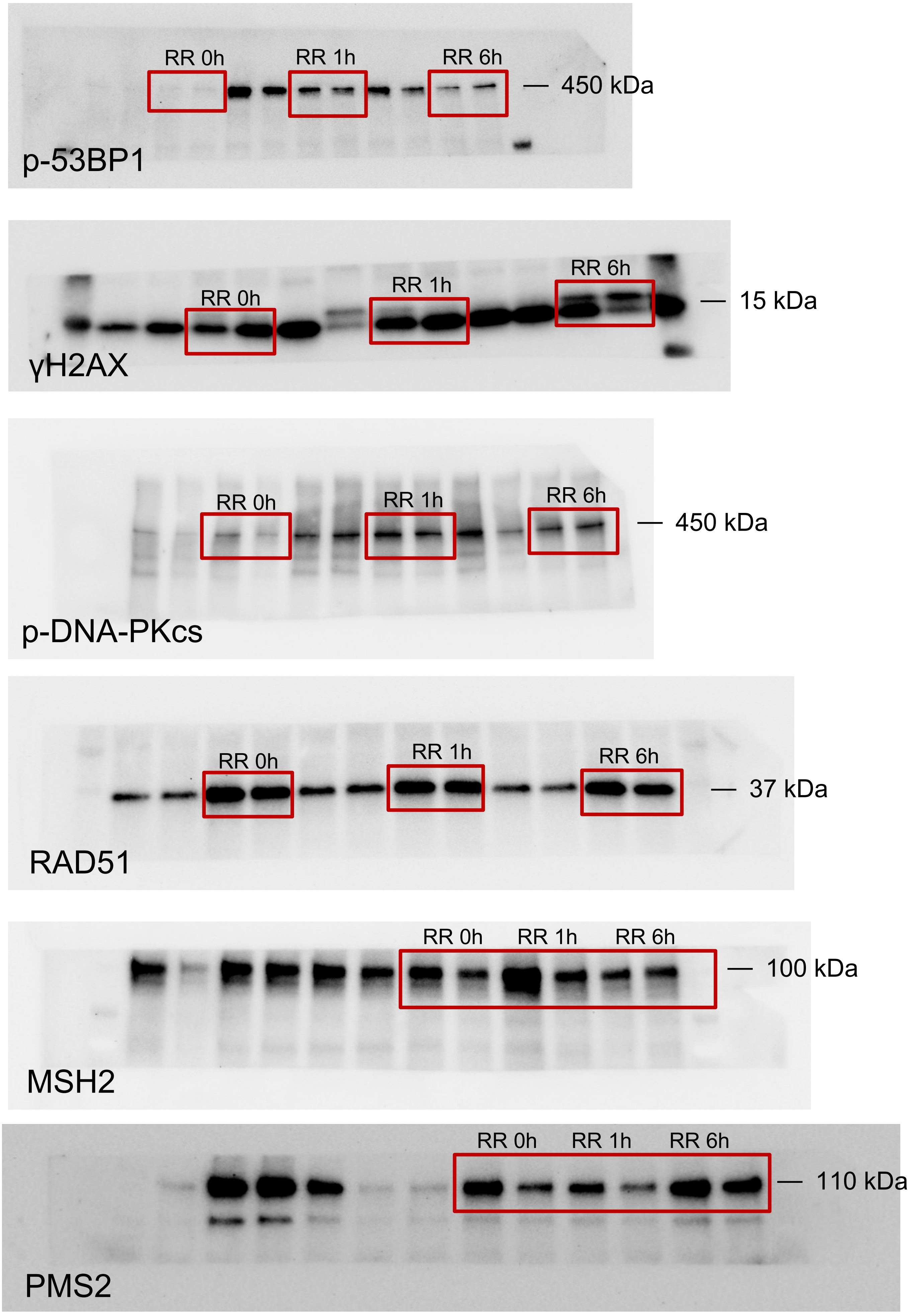

Figure 5 and Supplementary Figure S9 (22Rv1)

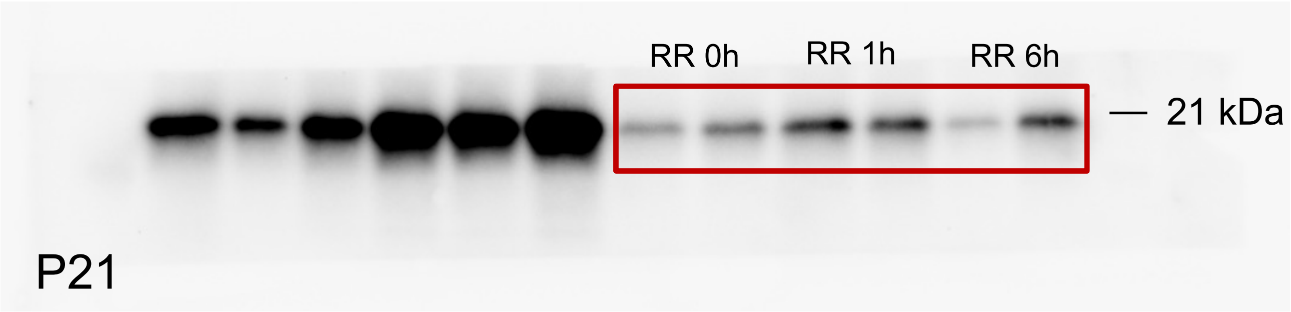

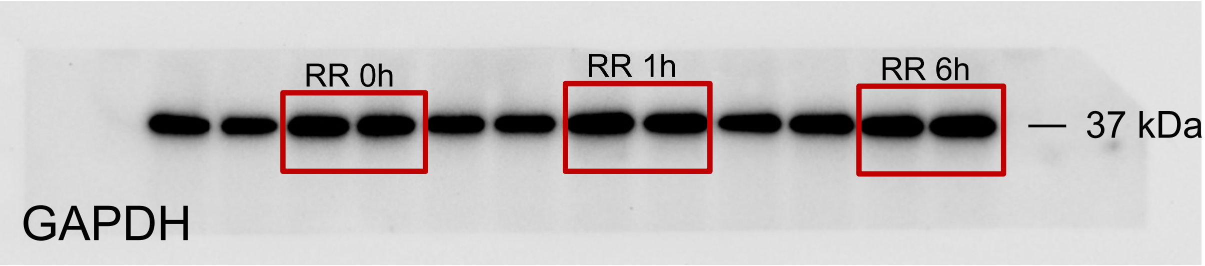

Figure 5 and Supplementary Figure S9 (FaDu)

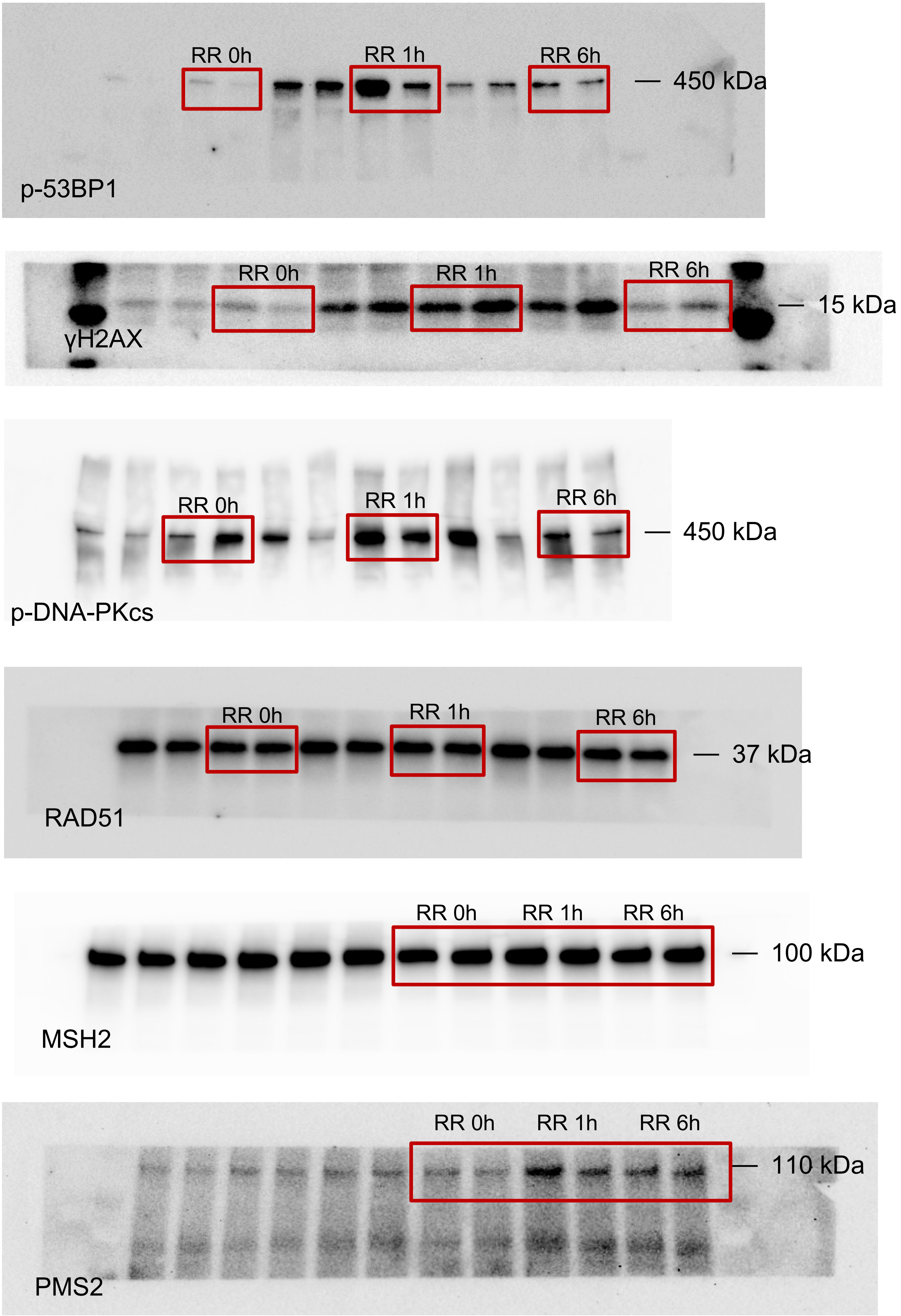

Figure 5 and Supplementary Figure S9 (FaDu)

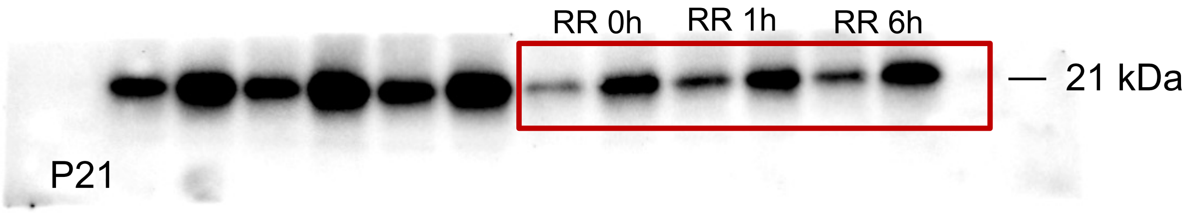

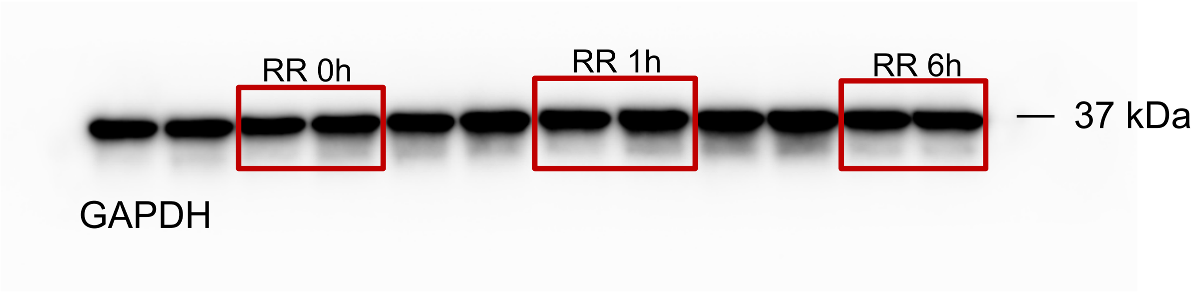

Supplement: Supplementary file 2 — Western Blot Original [file 41419_2024_7316_MOESM2_ESM.pdf]
